# Integrated Screening Identifies Elite Indigenous *Bacillus* Strains for Enhancing Wheat Productivity and Irrigation Water-Use Efficiency Under Full and Deficit Irrigation

**DOI:** 10.3390/life16091511

**Published:** 2026-09-10

**Authors:** Mohammed AI-dakhiI, Ahmed Abdelrahim, Abrar Felemban, Majed Alotaibi, Yaser Hassan Dewir, Medhat Rehan, Fahad Alotaibi, Salah El-Hendawy

**Affiliations:** 1 The National Research and Development Center for Sustainable Agriculture (Estidamah), Riyadh 11422, Saudi Arabia; maldakhil@estidamah.gov.sa (M.A.-d.); abdelrahima@estidamah.gov.sa (A.A.); afelemban@estidamah.gov.sa (A.F.); 2 Plant Production Department, College of Food and Agriculture Sciences, King Saud University, Riyadh 11451, Saudi Arabia; malotaibia@ksu.edu.sa (M.A.); ydewir@ksu.edu.sa (Y.H.D.); 3 Department of Plant Production, College of Agriculture and Food, Qassim University, Buraydah 51452, Saudi Arabia; m.rehan@qu.edu.sa; 4 Department of Soil Science, College of Food and Agriculture Sciences, King Saud University, Riyadh 11451, Saudi Arabia; fanalotaibi@ksu.edu.sa

**Keywords:** arid conditions, biostimulants, PGPR, PGP traits, sustainable agriculture, grain yield

## Abstract

Water scarcity is a major constraint to wheat production in arid regions, highlighting the need for sustainable approaches to improve crop productivity and irrigation water-use efficiency (IWUE). Although *Bacillus*-based bioinoculants have been widely investigated, the potential of indigenous strains adapted to arid environments remains underutilized for the developing of site-specific bioinoculants. This study developed an integrated screening strategy combining plant growth-promoting (PGP) traits characterization, greenhouse evaluation, correlation analysis, and multivariate analyses to identify elite indigenous *Bacillus* strains capable of improving wheat performance under contrasting irrigation regimes. Fifty-one indigenous *Bacillus* strains representing 19 species, identified by 16S rRNA gene sequencing, were characterized for indole-3-acetic acid (IAA), ammonia (NH_3_), siderophore production, and potassium-solubilizing activity and subsequently evaluated in greenhouse conditions under full (FI) and deficit (DI) irrigation. Plant growth was assessed at 85 days after sowing, while yield and yield-related traits were evaluated at physiological maturity (130 DAS). Significant variation was observed among the strains in both PGP traits and their effects on wheat performance. Selected strains increased vegetative growth traits by 22.2–53.7% under FI and 16.4–49.2% under DI, while improving yield-related traits and IWUE by 24.7–52.3% and 14.7–67.2%, respectively, compared with the uninoculated control. Correlation analysis identified IAA production as the PGP trait most strongly associated with wheat growth, grain yield, and IWUE, followed by NH_3_ production, whereas siderophore production showed weak associations with most agronomic traits. Hierarchical cluster analysis and principal component analysis consistently identified *Bacillus cereus* A2, *B. pumilus* D2 and E3, and *B. safensis* D5 as the elite strains, while several additional indigenous strains also exhibited considerable potential under both irrigation regimes. This study demonstrates that the integrated screening approach enabled the identification of strain-level differences that could not be adequately captured by individual PGP traits alone, highlighting indigenous *Bacillus* strains as valuable resources for developing locally adapted bioinoculants to improve wheat productivity, IWUE, and drought resilience in arid and semi-arid agroecosystems.

## 1. Introduction

Wheat (*Triticum aestivum* L.) is one of the world’s most important cereal crops and a major stable food, contributing substantially to global calorie and protein supplies [1,2,3]. However, sustainable wheat production is increasingly threatened by climate change, soil degradation, drought stress, and excessive reliance on agrochemical fertilizers [4,5]. Among these constraints, drought is particularly critical in arid and semi-arid regions such as Saudi Arabia, where limited freshwater resources and harsh climatic conditions severely restrict agricultural productivity [6,7]. With continued population growth and increasing environmental pressures, global wheat production must increase by nearly 50% by 2050 to meet future food demands and ensure food security [8]. Therefore, improving wheat productivity while increasing irrigation water use efficiency (IWUE) is essential for sustainable production in water-limited environments.

Water-deficit stress adversely affects wheat growth and productivity by disrupting numerous morphological, physiological, biochemical, and molecular processes, including cell division and expansion, nutrient uptake, transpiration, photosynthesis, stomatal conductance, and plant–water relations [9,10,11]. Water deficit also disturbs cellular homeostasis by altering enzymatic activity, phytohormone balance, secondary metabolism, nutrient acquisition, osmotic adjustment, nitrogen and proline metabolism, and antioxidant defense systems [12,13,14,15]. Moreover, drought induced accumulation of reactive oxygen species (ROS) causes oxidative damage to cellular membranes, proteins, lipids, and nucleic acids, thereby impairing photosynthetic performance and overall plant growth [12,13,14,15]. The severity of these effects depends on the intensity, duration, and timing of water deficit, with tillering, anthesis, and grain-filling being the most sensitive growth stages [16,17]. Consequently, sustainable strategies that maintain productivity and improve IWUE under deficit irrigation are increasingly important.

To mitigate the adverse effects of water-deficit stress on crop productivity, various strategies have been developed, including genetic improvement, improved agronomic practices, water-saving irrigation, crop calendar adjustment, and the application of plant growth regulators, soil amendments, mulches, organic fertilizers, and seed-priming techniques [17,18,19,20,21]. Although these approaches can improve drought tolerance and water-use efficiency, some are labor-intensive, time-consuming, costly, or difficult to implement on a large scale, while others may raise environmental concerns. Therefore, there is an increasing need for practical, cost-effective, and environmentally sustainable strategies that can be readily integrated into agricultural systems to enhance crop drought tolerance and sustain crop productivity under water-limited conditions. Among these strategies, plant growth-promoting rhizobacteria (PGPR) have emerged as an environmentally sustainable strategy for enhancing crop productivity and resilience under water-limited conditions. PGPR can promote plant growth and productivity through complementary direct and indirect mechanisms, including nutrient mobilization and the production of phytohormone, such as indole-3-acetic acid (IAA), gibberellins, and cytokinins, which stimulate root development and enhance water and nutrient uptake [22,23,24]. In addition, some PGPR also contribute to stress tolerance through siderophore production, osmotic adjustment, antioxidant defense, and the formation of exopolysaccharides (EPS) and biofilm, which can facilitate root colonization and improve soil moisture retention [24,25,26,27,28].

Among PGPR, the genus *Bacillus* has attracted considerable attention because of its exceptional ecological adaptability, broad agricultural applications, and ability to withstand harsh environmental conditions, including drought, salinity, heat, and nutrient-poor soils. As Gram-positive, endospore-forming bacteria, *Bacillus* spp. can maintain long-term viability, have an extended shelf life, facilitate formulation, remain compatible with commercial biofertilizers, and persist effectively under field conditions, making them ideal candidates for sustainable agriculture in water-scarce environments [25,27,29,30,31]. *Bacillus* spp. can promote plant growth through multiple mechanisms, including nutrient solubilization, siderophore and ammonia production, phytohormone synthesis, and stimulation of root development, thereby potentially improving nutrient and water acquisition. They also enhance drought tolerance by producing EPS, biofilms, and volatile organic compounds (VOCs), which improve soil aggregation, rhizosphere water retention, and root colonization. In addition, *Bacillus* modulates phytohormonal signaling, promotes osmotic adjustment, strengthens antioxidant defense systems, maintains photosynthetic efficiency and membrane integrity, and reduces ROS-induced oxidative damage. Collectively, these complementary mechanisms make *Bacillus* spp. highly effective microbial biostimulants for improving crop productivity, water-use efficiency, and resilience under water-deficit conditions [23,27,28,31,32,33,34]. Accumulating evidence demonstrates that *Bacillus* spp. effectively improve plant growth, productivity, and water-use efficiency under both well-watered and drought conditions. Species such as *B. subtilis*, *B. velezensis*, *B. cereus*, and *B. rugosus* have enhanced root growth, biomass accumulation, chlorophyll content, nutrient uptake, and grain yield in diverse crops, including wheat, rice, maize, soybean, sugarcane, sunflower, and pea [22,27,32,33,34]. Under water-deficit conditions, *Bacillus* inoculation further improves root architecture, plant water status, photosynthetic performance, antioxidant capacity, and nutrient acquisition, leading to greater biomass production, higher yield, and improved water-use efficiency [22,27,32,33,34,35]. These consistent findings highlight the considerable potential of *Bacillus* spp. as sustainable microbial biostimulants for enhancing crop productivity and IWUE under contrasting water regimes.

Despite the increasing use of *Bacillus*-based bioinoculants to improve crop performance under drought stress, the effectiveness of indigenous *Bacillus* strains originating from arid agricultural environments in simultaneously enhancing wheat productivity and IWUE under contrasting irrigation regimes remains comparatively underexplored. Commercial *Bacillus*-based biofertilizers are commonly formulated from species such as *B. subtilis*, *B. amyloliquefaciens*, *B. pumilus*, *B. licheniformis*, *B. megaterium*, and *B. velezensis* [35]. However, strains developed for commercial applications may not necessarily exhibit the same ecological fitness, rhizosphere competitiveness, or performance as locally adapted strains when introduced into environmentally distinct agroecosystems. This highlights the importance of identifying indigenous strains that are naturally associated with local agricultural environments and may possess plant growth-promoting (PGP) characteristics suited to water-limited conditions. Moreover, screening strains solely on the basis of PGP traits under laboratory conditions may not reliably predict their effects on plant growth, productivity, or IWUE under greenhouse conditions. An integrated evaluation that combines microbial PGP characteristics with plant performance under full and deficit irrigation is therefore needed to identify strains with consistent and agronomically relevant benefits. Therefore, this study aimed to isolate, identify, and characterize indigenous *Bacillus* strains from local agricultural rhizospheres, evaluate their key PGP traits, and systematically assess their ability to improve wheat growth, productivity, and IWUE under full and deficit irrigation. We hypothesized that locally adapted *Bacillus* strains exhibiting superior PGP traits, particularly IAA and NH_3_ production, would enhance wheat growth and productivity under FI and DI by promoting root development and improving water and nutrient acquisition. We further hypothesized that these beneficial effects would alleviate the adverse effects of water-deficit stress and improve IWUE under DI, and that an integrated screening approach combining PGP traits and plant responses would identify strains with superior and consistent performance across irrigation regimes.

## 2. Materials and Methods

### 2.1. Isolation of Bacillus Strains

The *Bacillus* strains evaluated in this study were isolated from the rhizosphere of wheat, date palm, and wild plants, such as Rimth (*Haloxylon salicornicum*) and Samar (*Acacia tortilis*) growing in the Al-Qassim region, Saudi Arabia, an arid environment characterized by high temperatures, low annual rainfall, and limited water availability. Rhizosphere soil samples were collected from healthy plants at depths of 5–20 cm from multiple agricultural sites in Buraydah (26°17′46.7″ N, 43°47′12.3″ E) and Unaizah (26°02′47.9″ N, 43°00′27.9″ E). Samples were placed in sterile zip-lock bags, transported to the laboratory at 4 °C, and processed immediately for microbial isolation.

*Bacillus* strains were isolated using serial dilution and spread-plate techniques. Briefly, 1–3 g of soil was suspended in 10 mL of sterile distilled water to separate and scatter rhizosphere-associated bacteria from the root surface before serially diluted to 10^−6^. Subsequently, 0.1 mL aliquots from the appropriate dilutions were spread onto sterile nutrient agar plates containing (*w*/*v*) 0.3% peptone, 0.5% NaCl, 0.3% yeast extract, and 1.5% agar, with the pH adjusted to 7.0. The plates were incubated at 37 °C for 24–48 h [36]. Colonies exhibiting typical *Bacillus*-like morphology were selected, and Gram-positive, rod-shaped isolates were purified by repeated streaking on fresh nutrient agar plates. A total of 51 isolates exhibiting vigorous growth and distinct colony morphology were preserved at −80 °C in LB medium supplemented with 40% (*v*/*v*) glycerol for subsequent characterization and evaluation.

### 2.2. Identification of Putative Bacillus Isolates

The putative bacterial isolates were identified based on partial sequencing of the 16S ribosomal RNA (16S rRNA) gene. Genomic DNA was extracted following the rapid extraction method described by Cook and Meyers [37] with slight modifications. Briefly, bacterial isolates were cultured in nutrient broth (NB) medium and incubated at 30 °C on a rotary shaker at 150 rpm for 24 h. Bacterial cells were harvested by centrifugation at 12,000 rpm for 3 min, washed once with Tris–EDTA (TE) buffer (pH 7.7), and resuspended in 300 µL of the same buffer. Cell lysis was achieved by heating the suspension at 95 °C for 10 min in a boiling water bath (ThermoMixer^®^ C, Eppendorf, Hamburg, Germany) followed by immediate cooling at room temperature. The lysate was then centrifuged at 12,000 rpm for 5 min, and the supernatant containing genomic DNA was transferred to sterile microtubes and stored at 4 °C until PCR analysis. DNA concentration and purity were assessment using a NanoDrop ND-1000 spectrophotometer (Thermo Scientific, Wilmington, DE, USA). Amplification of the 16S rRNA gene was performed using DreamTaq PCR Master Mix (2×) in 30 µL reaction volumes with the universal bacterial primers: forward primer was 27F (5′-AGAGTTTGATCATGGCTCAG-3′) and reverse primer was 1492R (5′-TACGGTTACCTTGTTACGACTT-3′). The thermal cycling conditions were as follows: an initial denaturation at 95 °C for 2 min, followed by 35 cycles of denaturation at 95 °C for 60 s, annealing at 45 °C for 30 s, and extension at 72 °C for 1.5 min, with a final extension at 72 °C for 7 min. The amplified PCR products were separated by electrophoresis on 1% agarose gel (*w*/*v*) stained with ethidium bromide to verify the presence of the expected DNA fragments. PCR products were subsequently purified and sequenced using Capillary Electrophoresis Sequencing (ABI 3730xl system) at Macrogen Inc. (Seoul, Republic of Korea). Forward and reverse sequences were assembled and edited using BioEdit software (version 7.7.1) to obtain consensus sequences. The resulting sequences were compared with those available in the NCBI GenBank database using the Basic Local Alignment Search Tool for nucleotides software (BLASTn, v2.16.0) for taxonomic identification and accession number assignment.

### 2.3. In Vitro Assessment of Plant Growth-Promoting Traits of Bacillus Strains

The *Bacillus* strains were evaluated in vitro, with three replicates per strain, for indole-3-acetic acid (IAA), ammonia, siderophore production, and potassium-solubilizing activity. IAA production was quantified colorimetrically following the method of Gang et al. [38]. Briefly, 0.2 mL of a three-day-old bacterial culture (10^7^–10^8^ CFU mL^−1^), growing in nutrient broth medium at 30 °C with shaking at 150 rpm, was inoculated into 20 mL of nutrient broth supplemented with 0.2% L-tryptophan and incubated at 30 °C for 48 h under shaking conditions, with uninoculated medium serving as the negative control. After centrifugation at 10,000 rpm for 5 min, 0.1 mL of the supernatant was mixed with 0.1 mL of Salkowski’s reagent in a 96-well plate, incubated in the dark for 30 min, and the absorbance was measured at 530 nm using a spectrophotometer plate reader (EPOCH2TS, BioTek, Winooski, VT, USA). IAA concentration (µg mL^−1^) was determined from a standard curve prepared with pure IAA.

Ammonia production was determined following the method of Chaudhary et al. [39] with slight modifications. Briefly, 100 µL of a bacterial preculture was inoculated into 20 mL of sterile peptone water medium containing 10 g/L of peptone (pH 7.2) and incubated at 30 °C for 48 h under shaking conditions. After centrifugation at 10,000 rpm for 5 min, 20 µL of the supernatant was mixed with 180 µL of Nessler’s reagent in a 96-well plate. The development of a yellow-to-brown color indicated positive ammonia production. Absorbance was measured at 450 nm using the same spectrophotometer plate reader, and ammonia concentration (mg mL^−1^) was determined from a standard curve prepared with ammonium hydroxide.

Siderophore production was qualitatively assessed using the Chrome Azurol S (CAS) agar assay described by Schwyn and Neilands [40]. Briefly, in a 9 cm sterile Petri dishes, fresh bacterial cultures were spot-inoculated onto CAS agar plates containing the CAS/Fe^3+^/hexadecyltrimethylammonium bromide indicator complex and incubated at 30 °C for 48–72 h. The formation of an orange or yellow halo around the colonies, resulting from the removal of Fe^3+^ from the blue CAS dye complex, was considered indicative of siderophore production, whereas the absence of a color change was recorded as a negative result.

Potassium-solubilizing activity was qualitatively assessed following the method of Yaghoubi et al. [41]. Briefly, bacterial strains were spot-inoculated onto Aleksandrov agar medium containing potassium feldspar (2%) as the insoluble potassium source and incubated at 30 °C for 72 h. The formation of a clear halo around the colonies was considered indicative of potassium solubilization.

### 2.4. Preparation of Bacillus Strain Inoculum

The bacterial inoculum for the pot experiment was prepared by transferring a loopful of each freshly grown *Bacillus* stock culture into 20 mL of nutrient broth (NB) and incubating the cultures at 30 °C for 48 h in a shaking incubator at 150 rpm. Wheat seeds were soaked overnight in the bacterial suspension supplemented with 2% sucrose as an adhesive. A second inoculum for plant inoculation was prepared by transferring 0.5 mL of the freshly grown culture into 150 mL of sterile NB medium and incubating it at 30 °C for 48 h at 150 rpm. Bacterial population density was estimated spectrophotometrically at 600 nm and adjusted to approximately 1 × 10^8^–5 × 10^8^ CFU mL^−1^. Before plant inoculation, the bacterial suspensions were diluted to a final volume of 250 mL and supplemented with 5 g sucrose as an adhesive and carbon source, resulting in final cell densities of 6 × 10^7^–3 × 10^8^ CFU mL^−1^.

### 2.5. Greenhouse Evaluation of Bacillus Strains Under Full and Deficit Irrigation

#### 2.5.1. Greenhouse Experimental Setup

**Figure 1:**
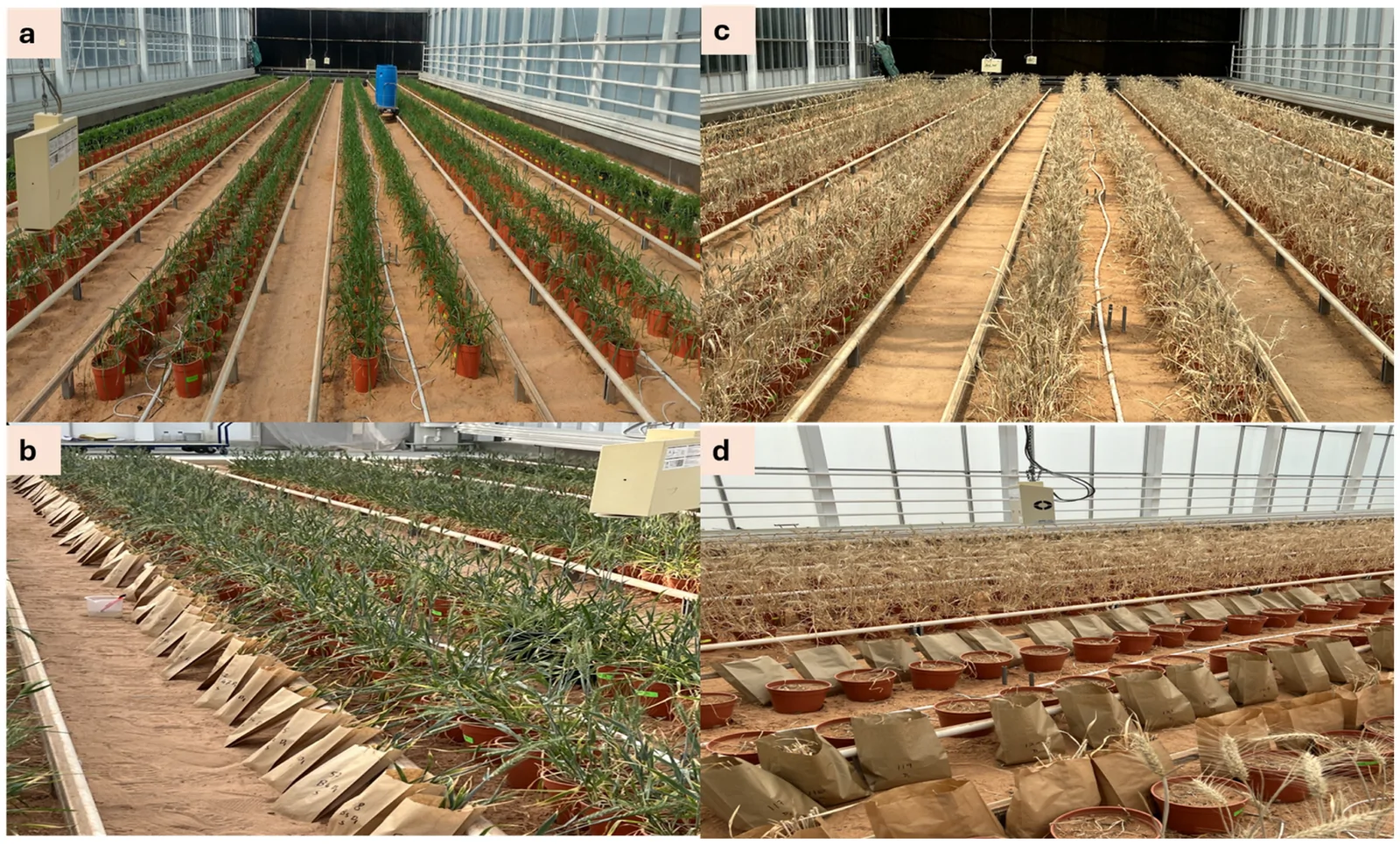
Greenhouse experimental setup and plant sampling during the wheat growth cycle. (**a**) wheat plants during the vegetative growth stage; (**b**) plant sampling at the vegetative growth stage; (**c**) wheat plants at physiological maturity; and (**d**) plant sampling at physiological maturity.

The pot experiment was conducted during the 2024/2025 winter growing season in a mid-tech greenhouse (Figure 1) at the National Research and Development Center for Sustainable Agriculture (Estidamah), Riyadh, Saudi Arabia. The greenhouse was constructed with tempered glass panels providing 91% light transmission, 80% hemispherical light transmission, and 75% fog transmission. Environmental conditions were continuously monitored using a network of distributed sensors integrated with a computerized climate-control system. Throughout the 130-day experimental period, the system maintained day/night temperatures of 25/18 °C and relative humidity levels of approximately 70%/50%, respectively.

Each *Bacillus* strain was evaluated in 20 cm diameter × 18 cm height plastic pots (Figure 1) filled with 10 kg of air-dried, autoclaved soil passed through a 2 mm sieve. The soil was sandy (91.3% sand, 7.6% silt, and 1.1% clay), with an electrical conductivity (EC) of 1.1 dS m^−1^, a pH of 7.83, 3.07 g kg^−1^ organic carbon, and available N, P, K, Ca, and Mg concentrations of 28.2, 2.32, 21.0, 7.80, and 12.9 mg kg^−1^, respectively. Ten inoculated seeds of the wheat cultivar ‘Gemmeiza-8’ were sown in each pot and thinned to six uniform seedlings 10 days after emergence. A second inoculation was applied 25 days after sowing by adding approximately 50 mL of the corresponding *Bacillus* suspension around the root zone of each pot to promote effective rhizosphere colonization. The pots were periodically re-randomized inside the greenhouse throughout the experimental period to minimize microenvironmental gradients and positional effects.

#### 2.5.2. Experimental Design and Drought Treatment

The pot experiment was arranged in a randomized complete block design (RCBD) with a split-plot layout and three replicates. The two irrigation regimes, full irrigation (FI) and deficit irrigation (DI), were assigned to the main plots, whereas the 51 *Bacillus* strains and the uninoculated control were randomly assigned to the subplots. Irrigation treatments were initiated 30 days after sowing by maintaining soil moisture at 80–100% and 40–50% of field capacity under FI and DI, respectively. Field capacity was determined gravimetrically before treatment application following the method of Earl [42], which involved saturating the soil and allowing excess gravitational to drain freely for 48 h. Throughout the experiment, pots were weighed every 1–2 days using a digital balance (30 kg capacity, ±5 g accuracy), and water lost through evapotranspiration was replenished to maintain the target FC for each irrigation treatment. Irrigation volumes were adjusted periodically to account for increasing plant biomass during growth.

#### 2.5.3. Plant Sampling and Measurements

Plant sampling was performed twice: at 85 days after sowing to assess vegetative growth and at physiological maturity (130 days after sowing) to evaluate yield and yield-related traits. At the first sampling, two plants per pot were harvested to measure plant height (PH, cm), tiller number (TN), leaf number (LN), total leaf area (TLA, cm^2^), total fresh weight (TFW, g), and total dry weight (TDW, g). Plant height was measured from the soil surface to the spike tip, TFW was recorded immediately after harvest, and TLA was determined using a leaf area meter (LI-3100; LI-COR Inc., Lincoln, NE, USA). Plant samples were then oven-dried at 75 °C to constant weight for TDW determination.

At physiological maturity (Figure 1), the remaining four plants per pot were harvested to determine spike number (SpN), spike weight (SpW, g), seed number (SN), grain yield (GY, kg m^−2^), and biological yield (BY, kg m^−2^). GY and BY were initially measured on a per-plant basis and converted to kg m^−2^ using the pot surface area (0.0314 m^2^). Harvest index (HI, %) was calculated as the ratio of GY to BY. Irrigation water-use efficiency (IWUE, kg m^−3^) was calculated by dividing GY by the total irrigation water applied, which was 12.00 and 5.85 L pot^−1^ under FI and DI, respectively.

### 2.6. Data Analysis

Data for all measured plant traits were analyzed using two-way analysis of variance (ANOVA) based on the general linear model, with irrigation regime, *Bacillus* strain, and their interaction treated as fixed effects. When significant effects were detected, treatment means were separated using Tukey’s honestly significant difference (HSD) test at *p* ≤ 0.05. Statistical analyses were performed using Statistix 9 (Analytical Software, Tallahassee, FL, USA). Before analysis, the assumptions of normality and homogeneity of variance were verified. Multivariate analyses, including hierarchical cluster analysis (HCA) and principal component analysis (PCA), were performed to classify *Bacillus* strains and examine their relationships with the measured plant traits. Pearson’s correlation analysis was used to evaluate associations between PGP traits and measured plant traits across irrigation regimes. Correlation analysis, HCA, and PCA were conducted using R version 4.0.2 in the RStudio desktop environment (version 2025.9.1.494).

## 3. Results

### 3.1. Molecular Identification of Isolates Using the Sequencing of 16S rRNA Gene

Molecular identification based on 16S rRNA gene sequencing confirmed that all 51 bacterial isolates belonged to the genus *Bacillus*, with sequence similarities ranging from 91.49% to 100% relative to reference sequences in the NCBI database (Table 1). The isolates were assigned to 19 *Bacillus* species, demonstrating substantial taxonomic diversity. *Bacillus subtilis* was the predominant species (18 strains), followed by *B. amyloliquefaciens* (10 strains). *B. safensis* and *B. halotolerans* were each represented by three strains, whereas *B. cereus* and *B. pumilus* each comprised two strains. The remaining 13 species, including *B. crassostreae*, *B. inaquosorum*, *B. tequilensis*, *B. sonorensis*, *B. atrophaeus*, *B. aerophilus*, *B. thuringiensis*, *B. rugosus*, *B. firmus*, *B. stratosphericus*, *B. altitudinis*, *B. methylotrophicus*, and *B. aryabhattai*, were each represented by a single isolate. Several isolates, including *B. pumilus* D2, *B. halotolerans* B14, *B. safensis* D1, multiple *B. amyloliquefaciens* strains (Inh10, B18, F2, and Inh8), and several *B. subtilis* strains (e.g., B19, E4, and Inh1), exhibited 100% sequence similarity to their corresponding reference strains, indicating a high degree of genetic relatedness. Otherwise, isolates with relatively low sequence similarity values were described as strains closely related to their nearest reference taxa retrieved from the NCBI database rather than being treated as definitively identified species. Specifically, *Cytobacillus firmus* D4, closely related to *Cytobacillus firmus* MSF4 (PZ766227; 91.49% similarity); *Bacillus pumilus* E3 presented a close relation to *Bacillus pumilus* NWMCC0302 (CP141820; 93.63% similarity); *Bacillus cereus* AB4 was closely related to *Bacillus cereus* NRC2 (ON231813; 95.76% similarity); whereas *Bacillus atrophaeus* AB5 displayed relatively relation to *Bacillus atrophaeus* JCM 9070 (OQ876677; 93.33% similarity).

### 3.2. Phylogenetic Analysis

**Figure 2:**
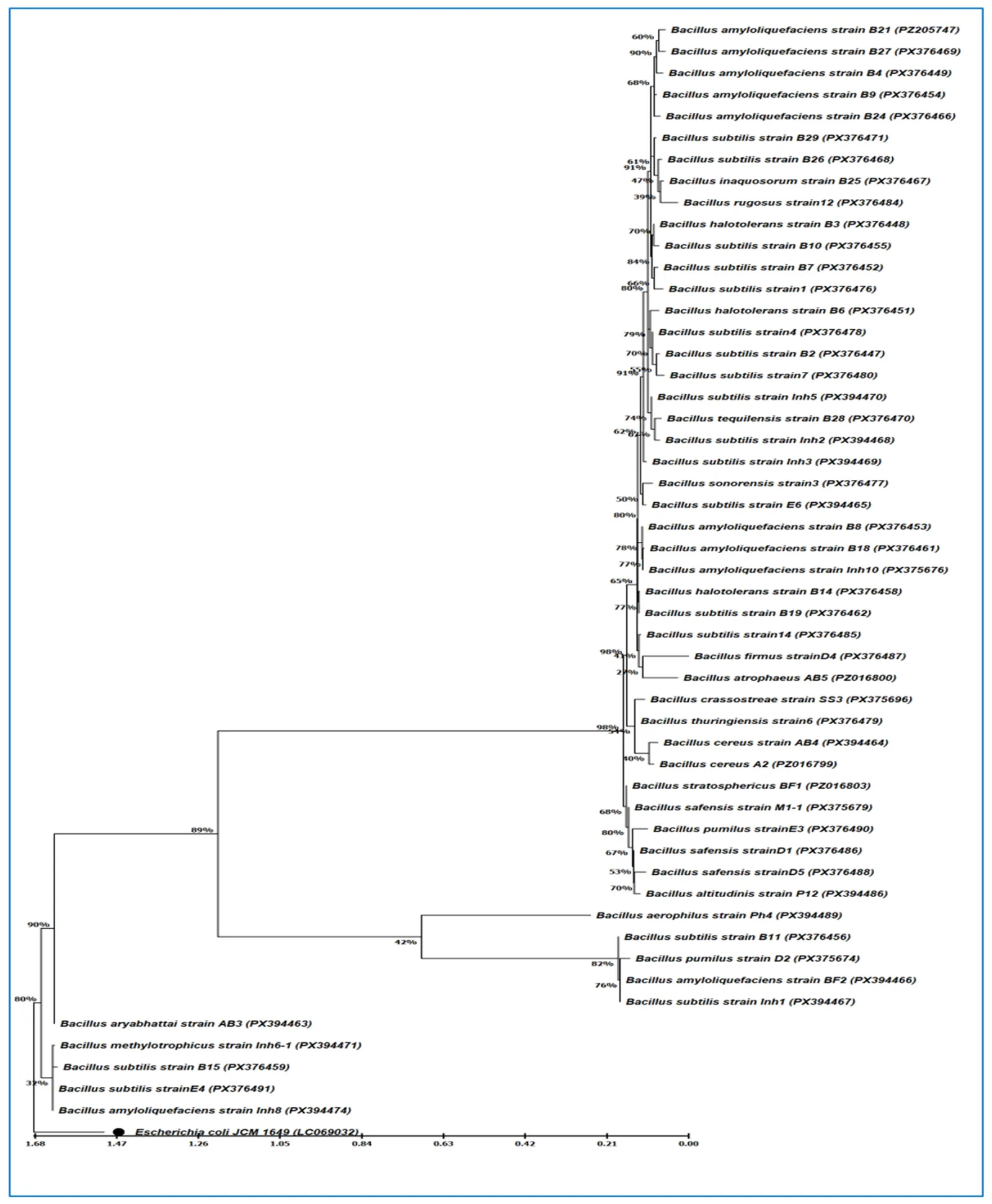
Phylogenetic tree based on 16S rRNA gene sequences showing the relationships among the *Bacillus* strains evaluated in this study. The tree was rooted using *Escherichia coli* JCM 1649 as the outgroup. Bootstrap values (%) based on 1000 replicates are shown at the branch nodes. The phylogenetic tree was constructed using the Neighbor-Joining method and validated by Maximum Likelihood analysis.

Phylogenetic analysis based on 16S rRNA gene sequences grouped the 51 isolates into several distinct clades within the genus *Bacillus* (Figure 2). The tree was rooted using *Escherichia coli* JCM 1649 as the outgroup, which was clearly separated from all *Bacillus* isolates. The largest clade comprised most *B. subtilis* and *B. amyloliquefaciens* isolates, indicating their close phylogenetic relationships. The *B. subtilis* isolates were further divided into several closely related subclades, reflecting intraspecific genetic diversity, whereas most *B. amyloliquefaciens* isolates clustered together with strong bootstrap support. Closely related species, including *B. halotolerans*, *B. sonorensis*, *B. tequilensis*, *B. inaquosorum*, and *B. rugosus* clustered adjacent to the *B. subtilis* and *B. amyloliquefaciens* lineage, whereas *B. cereus*, *B. firmus*, *B. thuringiensis*, *B. crassostreae*, *B. pumilus*, *B. safensis*, *B. atrophaeus*, *B. altitudinis*, *B. methylotrophicus*, and *B. aryabhattai* formed distinct branches, demonstrating the broad genetic diversity of the indigenous isolates. Notably, the most effective plant growth-promoting strains identified in this study, including *B. cereus* A2, *B. pumilus* D2 and E3, and *B. safensis* D5, were distributed across different phylogenetic lineages rather than a single cluster, suggesting that superior plant growth-promoting potential is not restricted to a particular taxonomic group within the genus *Bacillus*. Overall, the phylogenetic analysis revealed substantial taxonomic diversity among the indigenous *Bacillus* isolates, providing a broad genetic resource for identifying strains with superior PGP traits and drought-mitigation potential.

### 3.3. Evaluation of Plant Growth-Promoting Traits of Bacillus Strains

The 51 *Bacillus* strains were screened in vitro for four PGP traits: IAA, NH_3_, and siderophore production, as well as potassium-solubilizing activity (Table 1). All strains produced IAA in the presence of L-tryptophan, although concentrations varied markedly, ranging from 8.77 to 159.96 µg mL^−1^, demonstrating substantial metabolic diversity. The highest IAA production was recorded in *B. pumilus* D2, followed by *B. cereus* A2 and *B. crassostreae* SS3. Relatively high IAA production was also observed in *B. thuringiensis* S-6, *B. subtilis* E4, and *B. pumilus* E3, whereas *B. aerophilus* Ph4 and *B. atrophaeus* AB5 produced the lowest amounts (Table 1). Ammonia production also varied considerably among the strains, ranging from 0.01 to 16.19 mg L^−1^. The highest NH_3_ production was observed in *B. cereus* A2, *B. safensis* D5, and *B. pumilus* D2, whereas *B. aerophilus* Ph4 and *B. atrophaeus* AB5 exhibited negligible production. Several *B. subtilis* strains (S-1, S-4, and S-7) and *B. pumilus* E3 also showed relatively high NH_3_ production (Table 1). Siderophore production was detected in 47 of the 51 strains (92.2%), with production ranging from low (+) to very high (+++). Eleven strains exhibited strong siderophore production (+++), whereas four strains (*B. pumilus* D2, *B. crassostreae* SS3, *B. subtilis* Inh1, and *B. atrophaeus* AB5) showed no detectable activity (Table 1). Potassium-solubilizing activity was detected in only three strains (5.9%): *B. cereus* A2, *B. amyloliquefaciens* B18, and *B. rugosus* S-2. Overall, the substantial variation observed among the indigenous *Bacillus* isolates demonstrates considerable diversity in their PGP potential, providing a valuable resource for selecting superior strains for subsequent greenhouse evaluation.

### 3.4. Analysis of Variance (ANOVA) of Different Measured Traits

Table S1 shows that both irrigation regime and *Bacillus* strain had significant (*p* ≤ 0.05) to highly significant (*p* ≤ 0.01) effects on all measured traits, except TN, which was not significantly affected by irrigation regime. Moreover, the significant irrigation × *Bacillus* strain interactions observed for all traits indicate that the effects of bacterial inoculation depended on the irrigation regime, reflecting differences among strains in their ability to enhance wheat growth, yield, and IWUE under FI and DI.

### 3.5. Impacts of Studied Treatments on Growth- and Yield Related Traits

Deficit irrigation significantly reduced all growth-related traits measured 85 days after sowing compared with FI, with PH being the least affected trait (Table 2). Across all *Bacillus* strains, DI reduced PH, TN, LN, TLA, TFW, and TDW by 13.1%, 21.8%, 36.4%, 61.3%, 36.6%, and 41.7%, respectively. Overall, trait sensitivity to water deficit followed the order: TLA > TDW > TFW > LN > TN > PH. The irrigation regime also markedly affected yield-related traits and IWUE (Table 3 and Table 4). Across all *Bacillus* treatments, DI reduced SpN, SpW, SN, GY, and BY by 42.1%, 39.5%, 34.5%, 32.2%, and 39.9%, respectively. In contrast, HI and IWUE increased by 13.4% and 39.1%, respectively, under DI compared with FI. Overall, the sensitivity of yield-related traits to water deficit followed the order: SpN > BY > SpW > SN > GY.

Significant variation was observed among the evaluated *Bacillus* strains for all growth-related traits under FI (Tables S2 and S3). *B. atrophaeus* AB5 was the most effective strain, producing the greatest PH (39.8%) and TFW (53.7%), while also ranking among the best strains for LN (25.0%) (Table 3). Other superior strains included *B. safensis* D5, which markedly increased TLA (37.8%), TFW (46.4%), and TDW (33.3%); *B. cereus* A2, which produced the greatest TLA (38.0%), TFW (28.1%), and TDW (36.7%); *B. pumilus* E3, which increased TLA (32.0%), TFW (25.2%), and TDW (35.6%); *B. subtilis* Inh5, which enhanced PH (20.8%), LN (22.2%), TLA (24.5%), TFW (42.5%), and TDW (25.5%); and *B. pumilus* D2, which improved TN (8.1%), TLA (28.1%), TFW (28.2%), and TDW (30.6%). Additional strains exhibited superiority for specific traits, including *B. aerophilus* Ph4, *B. aryabhattai* AB3, *B. altitudinis* P12, and *B. cereus* AB4, which increased PH by 27.9%, 25.5%, 24.3%, and 24.0%, respectively. Likewise, *B. rugosus* S-12 improved LN (25.9%) and TLA (27.8%), *B. subtilis* E6 increased LN by 29.6%, *B. subtilis* Inh1 and Inh2, and *B. amyloliquefaciens* Inh8 increased TFW by 36.2%, 36.7%, and 32.2%, respectively. In addition, *B. crassostreae* SS3 increased TDW by 25.4%, relative to the uninoculated control (Table 3).

Significant variation was also observed among the evaluated *Bacillus* strains under DI (Tables S2 and S3). *B. subtilis* Inh2 was among the most effective strains, increasing PH, LN, and TFW by 33.0%, 42.1%, and 24.2%, respectively (Table 4). Likewise, *B. cereus* A2 consistently produced the greatest TLA (45.1%) and TDW (35.3%), while *B. pumilus* D2 markedly enhanced TLA (31.3%), TFW (19.9%), and TDW (30.1%). Other superior strains included *B. safensis* D1, which increased LN (47.4%) and TFW (23.0%); *B. thuringiensis* S-6, *B. firmus* D4, and *B. amyloliquefaciens* F2, which improved both TLA by 28.8%, 20.7%, and 20.5%, respectively, and TDW by 22.5%, 18.4%, and 17.4%, respectively. Additional strains exhibited superiority for specific traits, including *B. aerophilus* Ph4, *B. cereus* AB4, and *B. subtilis* E6, which increased PH by 40.2%, 37.3%, and 36.6%, respectively. *B. amyloliquefaciens* B18 and *B. halotolerans* B14 increased TN by 33.4% and 23.7%, respectively, whereas *B. halotolerans* B3, *B. altitudinis* P12, *B. rugosus* S-12, *B. amyloliquefaciens* B8, *B. stratosphericus* F1, and *B. subtilis* B10 enhanced LN by 49.2%, 45.6%, and 42.1% (the latter five strains), respectively. In addition, *B. subtilis* Inh5, *B. methylotrophicus* Inh6-1, *B. atrophaeus* AB5, and *B. crassostreae* SS3 improved TFW by 39.1%, 31.4%, and 20.6%, and TDW by 16.4%, respectively, relative to the uninoculated control (Table 4).

Significant variation was also observed among the evaluated *Bacillus* strains for all yield-related traits under FI (Tables S3 and S4). *B. cereus* A2 was the most effective strain, producing the greatest SN (40.8%), GY (36.9%), BY (49.7%), and IWUE (36.9%), while also ranking among the best strains for SpN (27.3%) and SpW (46.9%) (Table 3). Other superior strains included *B. safensis* D5, which increased SpN (38.2%), SpW (50.2%), SN (34.8%), GY (29.6%), BY (39.1%), and IWUE (29.6%); *B. pumilus* E3, which enhanced SpN (27.3%), SpW (52.3%), SN (35.2%), GY (34.5%), BY (31.6%), and IWUE (34.5%); and *B. pumilus* D2, which increased SpN (43.6%), SpW (30.4%), GY (30.0%), BY (33.1%), and IWUE (30.0%). Additional strains showed superiority for specific yield-related traits. *B. subtilis* Inh2 ranked among the best strains for SpN (27.3%), *B. amyloliquefaciens* Inh8 increased SpW by 37.2%, *B. crassostreae* SS3 enhanced SN and BY by 32.5% and 30.6%, respectively, whereas *B. subtilis* Inh5 increased SN and BY by 24.7% and 29.2%, respectively. In contrast to the other yield-related traits, the greatest improvements in HI were achieved by a different group of strains. *B. aryabhattai* AB3, *B. subtilis* B19, *B. halotolerans* B3, *B. amyloliquefaciens* B9, and *B. cereus* AB4 increased HI by 7.5%, 7.1%, 6.3%, 5.7%, and 5.6%, respectively, relative to the uninoculated control (Table 3).

Significant variation was also observed among the evaluated *Bacillus* strains for all yield-related traits under DI (Tables S3 and S4). *B. cereus* A2 was the most effective strain, producing the greatest SpN (27.3%), SN (67.2%), GY (44.7%), BY (78.5%), and IWUE (44.7%), while also ranking among the best strains for SpW (25.5%) (Table 4). Other superior strains included *B. pumilus* D2, which increased SpN (15.3%), SN (52.5%), GY (24.9%), BY (33.4%), and IWUE (24.9%); *B. firmus* D4, which enhanced SpN (27.3%), SpW (35.0%), GY (14.7%), and IWUE (14.7%); *B. thuringiensis* S-6, which increased SpW (26.0%), SN (35.9%), GY (17.7%), BY (19.8%), and IWUE (17.7%); and *B. crassostreae* SS3, which improved SpN (27.3%), SpW (23.0%), SN (33.8%), GY (18.9%), and IWUE (18.9%). Additional strains showed superiority for specific yield-related traits. *B. subtilis* Inh5 and *B. safensis* D5 increased BY by 21.0% and 18.6%, respectively, whereas *B. rugosus* S-12 and *B. amyloliquefaciens* B8 enhanced SpN by 18.2% and 15.3%, respectively. In contrast to the other yield-related traits, the greatest improvements in HI were achieved by a different group of strains. *B. subtilis* Inh1, *B. tequilensis* B28, *B. inaquosorum* B24, and *B. altitudinis* P12 increased HI by 12.7%, 10.1%, 8.7%, and 8.2%, respectively, relative to the uninoculated control (Table 4).

Overall, several *Bacillus* strains consistently improved wheat growth- and yield-related traits under both irrigation regimes. Among them, *B. cereus A2* and *B. pumilus D2* were the most effective, markedly enhancing multiple growth traits (TLA, TDW, and/or TFW) as well as yield-related traits (SpN, SpW, SN, GY, BY, and IWUE) under both FI and DI (Table 3 and Table 4). Likewise, *B. safensis* D5, *B. subtilis* Inh2, *B. subtilis* Inh5, and *B. crassostreae* SS3 consistently improved several growth- and/or yield-related traits across both irrigation regimes, while *B. rugosus* S-12 and *B. altitudinis P1*2 enhanced specific traits under both conditions. Under FI, *B. atrophaeus* AB5 and *B. pumilus* E3 exhibited superior performance by markedly improving multiple growth and yield-related traits, whereas *B. aerophilus* Ph4, *B. aryabhattai* AB3, *B. amyloliquefaciens* Inh8, *B. subtilis* B19, *B. amyloliquefaciens* B9, and *B. cereus* AB4 were particularly effective for specific growth or yield traits. Under DI, *B. firmus* D4, *B. thuringiensis* S-6, *B. safensis* D1, *B. amyloliquefaciens* F2, *B. methylotrophicus* Inh6-1, *B. amyloliquefaciens* B18, *B. halotolerans* B3 and B14, *B. amyloliquefaciens* B8, *B. stratosphericus* F1, *B. subtilis* B10, *B. tequilensis* B28, and *B. inaquosorum* B24 showed superior performance for one or more growth- or yield-related traits (Table 3 and Table 4).

### 3.6. Correlation Matrix

**Figure 3:**
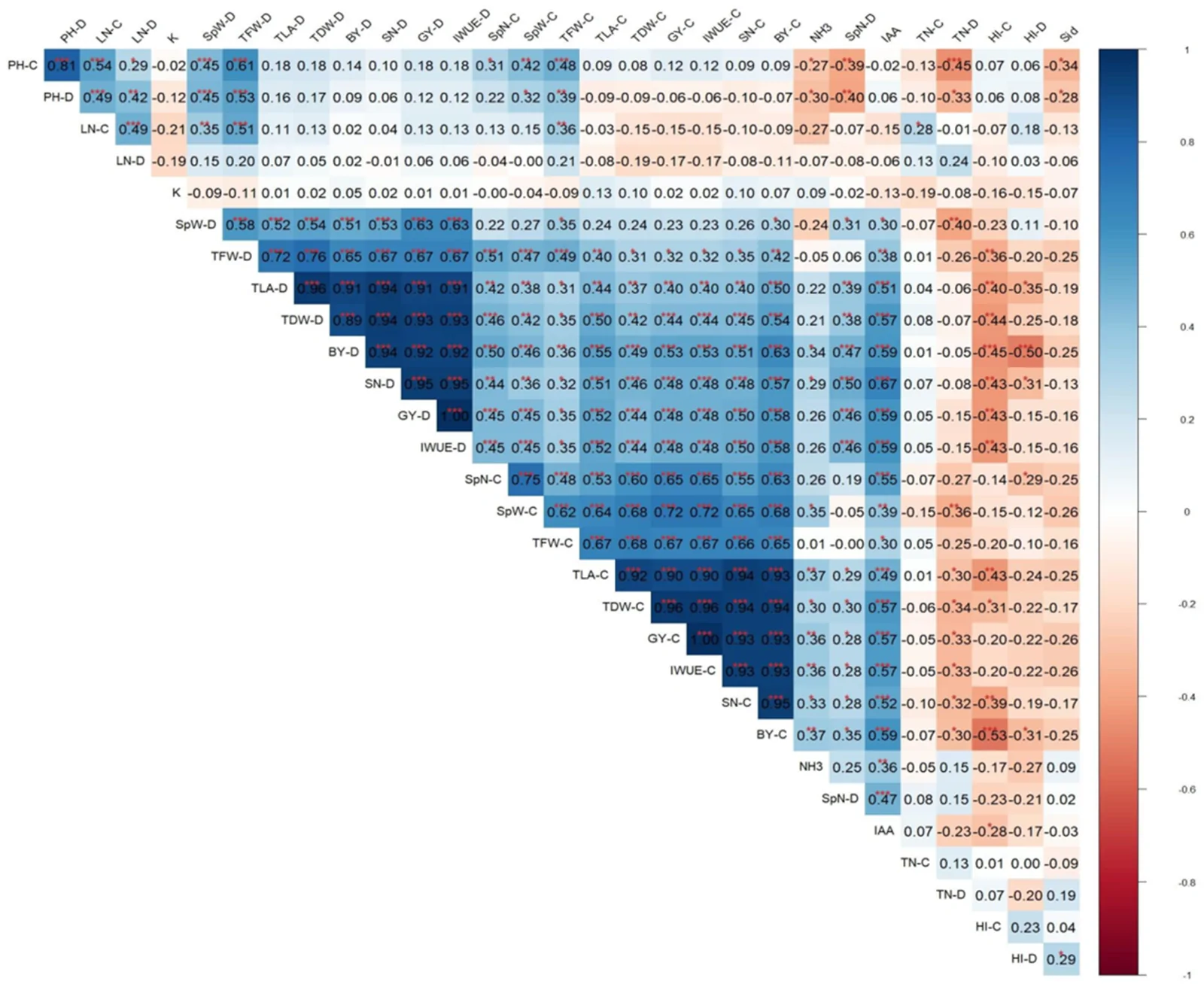
Pearson’s correlation coefficients (r) among growth- and yield-related traits measured under full irrigation (FI) and deficit irrigation (D), and plant growth-promoting (PGP) traits. PH, plant height (cm); TN, tiller number per plant; LN, leaf number per plant; TLA, total leaf area (cm^2^ plant^−1^); TFW, total fresh weight (g plant^−1^); TDW, total dry weight (g plant^−1^); SpN, spike number per plant; SpW, spike weight (g plant^−1^); SN, seed number per plant; GY, grain yield (kg m^−2^); BY, biological yield (kg m^−2^); HI, harvest index (%); IWUE, irrigation water-use efficiency (kg m^−3^); IAA, indole-3-acetic acid; NH_3_, ammonia; Sid, siderophore production; and K, potassium-solubilizing activity. Correlation coefficients (r) marked with *, **, and *** in the correlation matrix indicate statistical significance at *p* < 0.05, *p* < 0.01, and *p* < 0.001, respectively.

Among the evaluated PGP traits, IAA production showed the strongest positive associations with wheat performance under both irrigation regimes, with correlation coefficients ranging from *r* = 0.30–0.59 under FI and *r* = 0.30–0.67 under DI. The strongest relationships were observed with TLA, TDW, SN, GY, BY, and IWUE, particularly under DI (Figure 3). In comparison, NH_3_ production exhibited moderate positive associations (*r* = 0.26–0.37 under FI and *r* = 0.21–0.34 under DI), with the highest correlations involving TLA, TDW, SpW, SN, GY, BY, and IWUE under FI and BY and SN under DI. Conversely, siderophore production displayed weak relationships with most measured traits, with correlation coefficients ranging from *r* = −0.34 to 0.04 under FI and *r* = −0.28 to 0.29 under DI. The strongest negative association was observed with PH under both irrigation regimes, whereas HI exhibited a weak positive correlation under DI (*r* = 0.29). Likewise, potassium-solubilizing activity showed only weak and largely non-significant associations with wheat growth, yield, and IWUE, with correlation coefficients ranging from *r* = −0.21 to 0.10 under FI and *r* = −0.19 to 0.01 under DI (Figure 3).

Correlation analysis revealed strong positive relationships among vegetative growth traits, yield-related traits, and between growth and yield traits under both irrigation regimes (Figure 3). Among the vegetative traits, TLA, TFW, and TDW were strongly interrelated under both FI (*r* = 0.67–0.92 ***) and DI (*r* = 0.72–0.96 ***). Similarly, strong positive correlations were observed among yield-related traits. Under FI, GY was highly correlated with SN (*r* = 0.93 ***), BY (*r* = 0.93 ***), SpW (*r* = 0.72 ***), and SpN (*r* = 0.65 ***), while BY was strongly associated with SN (*r* = 0.95 ***), SpW (*r* = 0.68 ***), and SpN (*r* = 0.63 ***). A comparable pattern was observed under DI, where GY remained strongly correlated with SN (*r* = 0.95 ***), BY (*r* = 0.92 ***), SpW (*r* = 0.63 ***), and SpN (*r* = 0.46 ***), whereas BY was closely associated with SN (*r* = 0.94 ***), SpW (*r* = 0.54 ***), and SpN (*r* = 0.47 ***). Correlations between vegetative growth and yield-related traits further identified TDW as the trait most closely associated with grain productivity, exhibiting the strongest positive correlations with GY (*r* = 0.96 *** under FI and 0.93 *** under DI), BY (*r* = 0.94 *** and 0.89 ***), SN (*r* = 0.94 *** under both FI and DI), and SpW (*r* = 0.68 *** and 0.54 ***). Likewise, TLA was strongly correlated with GY, BY, SpW, and SN under FI (*r* = 0.64–0.94 ***) and DI (*r* = 0.52–0.94 ***), whereas TFW showed moderate positive correlations with the same traits under FI (*r* = 0.62–0.67 ***) and DI (*r* = 0.58–0.67 ***). In contrast, PH, TN, LN, and HI exhibited comparatively weaker correlations with most growth- and yield-related traits under both irrigation regimes (Figure 3).

### 3.7. Classification of Bacillus Strains Based on Plant Growth-Promoting Traits and Wheat Performance

**Figure 4:**
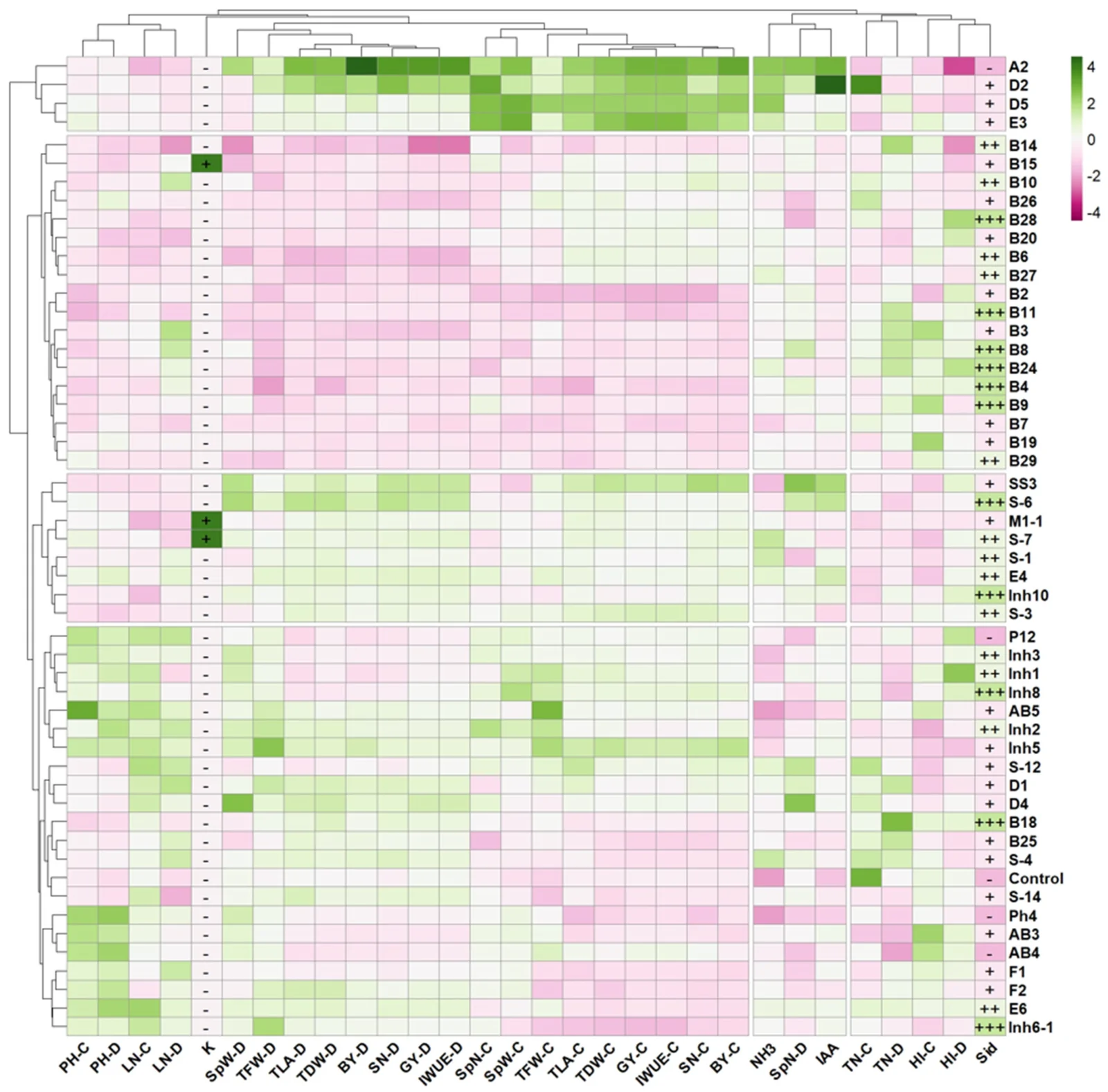
Hierarchical cluster analysis heatmap of *Bacillus* strains based on the mean values of wheat growth- and yield-related traits under both irrigation regimes and plant growth-promoting traits. The full names of *Bacillus* strain and trait abbreviations are presented in Table 1 and Table 2, respectively. For qualitative assays, “−” indicates no detectable activity, “+” indicates low activity, “++” indicates moderate activity, and “+++” indicates very high activity.

HCA and PCA were performed to classify the 51 *Bacillus* strains according to their PGP traits and wheat performance, as well as to examine the relationships between strains and measured traits. HCA grouped the strains into four distinct clusters (Figure 4). The means values of growth- and yield-related traits under full and deficit irrigation, together with plant growth-promoting (PGP) traits, for each *Bacillus* strain group identified by hierarchical cluster analysis based on the heatmap of all traits measured under both irrigation treatments and PGP traits are presented in Table 5. Group 1, comprising *B. cereus A2*, *B. pumilus D2* and E3, and *B. safensis D5*, represented the most superior cluster. These strains consistently produced the highest values for TLA, TFW, TDW, SpN, SpW, SN, GY, BY, and IWUE under both irrigation regimes and also exhibited the greatest PGP potential, with the highest mean IAA (95.76 µg mL^−1^) and NH_3_ (14.75 mg L^−1^) production (Figure 4; Table 5). Group 2, consisting of 18 strains, was the least effective cluster, exhibiting the lowest mean values for most growth- and yield-related traits and IWUE under both irrigation regimes. The only exceptions were HI under FI and TN under DI, where this group recorded the highest mean values. This group also had the lowest mean IAA production (29.72 µg mL^−1^), moderate NH_3_ production (7.71 mg L^−1^), and relatively high siderophore production. Group 3, comprising eight strains, ranked second for TLA, TDW, SN, GY, BY, and IWUE under both irrigation regimes, as well as for SpN and SpW under DI. It also exhibited the second-highest IAA (47.32 µg mL^−1^) and NH_3_ (8.55 mg mL^−1^) production, together with relatively high siderophore production. Group 4, comprising 21 strains together with the uninoculated control, ranked first for PH and TN under both irrigation regimes, as well as for LN under FI and HI under DI. It also ranked second for TFW under both irrigation regimes and for SpN, SpW, and HI under FI. This group exhibited relatively low IAA production (36.45 µg mL^−1^), relatively high siderophore production, and the lowest NH_3_ production (5.92 mg mL^−1^) (Figure 4; Table 5).

**Figure 5:**
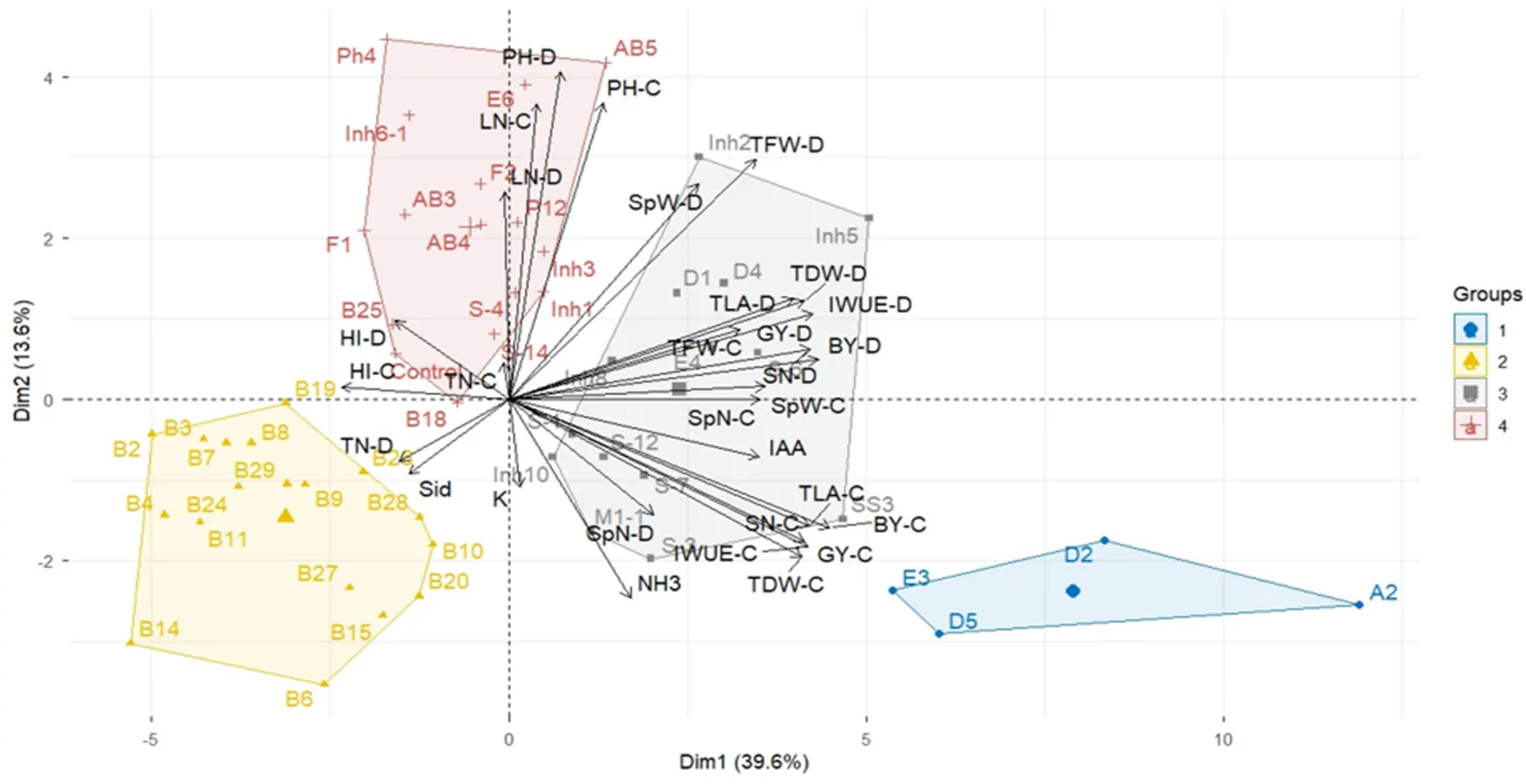
Principal component analysis biplot of *Bacillus* strains based on plant growth-promoting traits and wheat growth- and yield-related traits under full irrigation (FI, C) and deficit irrigation (DI, D). The full names of *Bacillus* strain and trait abbreviations are presented in Table 1 and Table 2, respectively.

PCA was performed to further characterize the relationships among *Bacillus* strains, PGP traits, and wheat performance under both irrigation regimes. The first two principal components explained 53.2% of the total variation, with PC1 and PC2 accounting for 39.6% and 13.6%, respectively (Figure 5), indicating that these components adequately summarized the major patterns of variation among the evaluated strains. PC1 was primarily associated with plant productivity and growth, showing high positive loadings for TLA, TFW, TDW, SpN, SpW, SN, BY, GY, and IWUE under both irrigation regimes (loading values = 0.153–0.259), together with IAA production (0.202) (Table S5). In contrast, HI, TN, potassium-solubilizing activity, and siderophore production exhibited negative or weak loadings on PC1. PC2 mainly represented morphological development, with the highest positive loadings for PH under FI (0.360) and DI (0.399), LN under FI (0.360) and DI (0.252), TFW and SpW under DI (0.291 and 0.262). In contrast, SpN under DI and NH_3_ production showed the largest negative loading on PC2 (−0.141 and −0.242) (Table S5).

The PCA biplot clearly separated the four HCA groups (Figure 5). Group 1 (blue polygon), comprising *B. cereus A2*, *B. pumilus D2* and E3, and *B. safensis D5*, was located on the positive side of PC1 and clustered closely with IAA, TLA, TFW, TDW, SN, GY, BY, and IWUE, confirming its superior plant growth-promoting capacity and performance under both irrigation regimes. Group 2 (yellow polygon) occupied the negative side of PC1 and was associated with lower values for most growth- and productivity-related traits. Group 3 (gray polygon) was positioned between Groups 1 and 2 and was closely associated with productivity-related traits under DI, indicating moderate performance and good drought adaptation. In contrast, Group 4 (red polygon) was distributed mainly in the upper region of the biplot near PH, LN, TN, and HI demonstrating stronger associations with plant morphological development than with grain productivity.

## 4. Discussion

In arid regions where water resources are increasingly limited, DI has become an important strategy for maximizing IWUE rather than crop yield per unit land area. In the present study, DI increased IWUE by 39.1% compared with FI, but this improvement was accompanied by substantial reductions in vegetative growth (TLA, TFW, and TDW) and yield-related traits (SpN, SpW, SN, GY, and BY), with decreases ranging from 32.2% to 61.3% (Table 2). These findings agree with previous reports showing that water-deficit stress can reduce wheat growth and productivity by 10–90%, depending on stress severity, duration, and crop developmental stage [13,14,16,17,43]. The marked reductions in growth and yield under DI are primarily attributed to impaired cell division and expansion, reduced photosynthetic capacity resulting from declines in chlorophyll content, photosystem II efficiency, Rubisco activity, and ATP and NADPH production, together with restricted carbon assimilation and biomass accumulation. Water deficit also limits nutrient uptake, particularly potassium, disrupts osmotic adjustment, hormonal balance, and antioxidant defense systems, and promotes excessive ROS accumulation, leading to oxidative damage, impaired assimilate production and translocation, and reduced reproductive development [11,12,13,15,17,44,45]. Thus, the increase in IWUE under DI should not be interpreted as an overall improvement in plant performance; rather, it reflects greater productivity per unit of water consumed despite a substantial reduction in absolute biomass and yield. This distinction is particularly important in arid production systems, where the objective is to minimize yield penalties while obtaining the greatest possible output from limited irrigation water. Collectively, these physiological and metabolic constraints explain the substantial decline in wheat growth and productivity observed under DI and provide a physiological basis for evaluating microbial strategies that can maintain plant performance while conserving irrigation water.

Although the agronomic strategies discussed in the Introduction can partially alleviate the adverse effects of water-deficit stress, their effectiveness is often limited because they target only one or a few drought-tolerance mechanisms [17,18,19,44,46]. Moreover, many of these approaches are labor-intensive, costly, or difficult to implement on a large scale. Consequently, PGPR has emerged as an environmentally sustainable and multifunctional strategy for enhancing crop growth, productivity, and drought tolerance. Unlike approaches that primarily modify one component of the plant–water system, PGPR may simultaneously influence root development, nutrient acquisition, hormonal signaling, rhizosphere conditions, and stress-defense responses. These complementary effects include improved water and nutrient acquisition, increased iron availability through siderophore production, phytohormone and NH_3_ synthesis, regulation of ABA- and ethylene-mediated signaling, enhanced rhizosphere water retention through exopolysaccharide (EPS) production, improved osmotic adjustment and root development, and strengthened antioxidant defense systems that reduce ROS-induced oxidative damage [47,48,49,50,51,52,53]. However, these mechanisms should not be assumed to operate to the same extent in all bacterial strains. The effectiveness of PGPR is strongly strain-dependent and depends on both the functional characteristics of the inoculant and its ability to establish a stable association with the host plant under the prevailing environmental conditions. Accordingly, indigenous PGPR may possess an ecological advantage in their native agroecosystems because they are already exposed to, and potentially adapted to, local soil, climatic, and rhizosphere conditions [35,54]. Previous studies have likewise shown that native *Bacillus* strains can enhance drought tolerance more effectively than some commercial inoculants, as reflected by greater proline accumulation and improved physiological performance in wheat [55,56]. This strain-specific adaptation provides a strong rationale for screening indigenous isolates rather than assuming that a broadly used commercial strain will perform optimally under all environmental conditions.

In the present study, indigenous *Bacillus* isolates recovered from the rhizosphere of healthy crops grown under arid conditions exhibited remarkable taxonomic and functional diversity, representing a valuable reservoir of locally adapted PGPR. The collection comprised 19 *Bacillus* species, dominated by *B. subtilis* and *B. amyloliquefaciens*, together with substantial variation in key PGP traits, including IAA, NH_3_, and siderophore production (Table 1; Figure 2). Such variability is expected because rhizosphere microbial communities are shaped by plant species and long-term adaptation to local soil and climatic conditions, resulting in strains with distinct ecological fitness and metabolic capabilities [54,55,56,57]. The universal ability of all isolates to produce IAA and NH_3_, together with the widespread occurrence of siderophore production, indicates that these traits may contribute to the overall plant-growth-promoting capacity of the indigenous isolates, but does not by itself establish that they are the predominant mechanisms operating in planta. Similar taxonomic and functional variation has been reported for *Bacillus* populations associated with crops grown in arid and semi-arid environments [24,25,27,33,58,59]. The marked functional variation among strains is particularly important because the presence of a PGP trait does not necessarily translate into equivalent plant benefits; therefore, direct evaluation of plant performance under the target irrigation conditions is essential for identifying agronomically effective strains.

The superior performance of several indigenous *Bacillus* strains in promoting wheat growth, yield, and IWUE under both FI and DI (Tables S2–S4, Table 3 and Table 4) was closely associated with their PGP profiles, particularly IAA and NH_3_ production. This interpretation is strongly supported by the correlation analysis, which identified IAA production as the PGP trait most closely associated with wheat performance. IAA exhibited the strongest and most consistent positive correlations with vegetative growth, biomass accumulation, GY, and IWUE, with correlation coefficients ranging from r = 0.30–0.59 under FI and r = 0.30–0.67 under DI (Figure 3). The stronger associations observed under DI indicate that auxin-related effects may become particularly relevant when water availability restricts root exploration and resource acquisition. A plausible mechanism is that bacterial IAA stimulates primary-root elongation and lateral root formation, increasing the volume of soil explored by the root system and thereby improving access to water and nutrients. Enhanced root development can subsequently support maintenance of plant water status and nutrient supply, which may help sustain photosynthesis, biomass accumulation, and reproductive development under water deficit [30,33,48,51,52,60,61,62,63,64]. However, because root architecture, plant water status, and endogenous hormone concentrations were not directly measured in the present study, these processes should be regarded as plausible mechanisms rather than experimentally confirmed causal pathways.

NH_3_ production also exhibited positive and significant correlations with most growth- and yield-related traits, although these relationships were generally moderate (r = 0.26–0.37 under FI and r = 0.21–0.34 under DI; Figure 3), suggesting that ammonia production contributed to improved wheat growth and productivity under both favorable and water-deficit conditions. The positive association may reflect the contribution of bacterial nitrogen-related activity to local nutrient availability, which could support chlorophyll formation, photosynthetic activity, and biomass accumulation. Nevertheless, the moderate correlation coefficients indicate that NH_3_ production alone cannot explain the variation in plant performance among the isolates, reinforcing the concept that multiple PGP functions operate simultaneously.

Although 92.2% of the evaluated *Bacillus* strains produced siderophores, their correlations with most growth- and yield-related traits were weak and generally non-significant (Figure 3). This finding indicates that, despite the well-established role of siderophores in enhancing iron availability for chlorophyll biosynthesis and photosynthetic electron transport, improving micronutrient acquisition, and alleviating abiotic stress [30,36,50,58,59,60,61,62,63], siderophore production was not the primary factor explaining the variation in plant performance among the tested strains. The high frequency of siderophore production may partly explain why this trait showed limited discriminatory power: when a functional trait is present in most isolates, differences in that trait are less likely to account for the large variation in plant performance among strains. Instead, siderophore activity likely acted synergistically with other PGP mechanisms, particularly IAA and NH_3_ production, as well as with additional mechanisms not evaluated in the present study. This finding highlights an important limitation of selecting bioinoculants solely on the basis of individual laboratory PGP traits. A strain that exhibits a strong PGP activity in vitro may not necessarily provide the greatest benefit to the plant under greenhouse or water-deficit conditions.

The plant responses observed following inoculation further support this multi-trait interpretation. Selected indigenous *Bacillus* strains increased vegetative growth traits by 22.2–53.7% under FI and 16.4–49.2% under DI, while enhancing yield-related traits and IWUE by 24.7–52.3% and 14.7–67.2%, respectively, relative to the uninoculated control (Table 3 and Table 4). Rather than interpreting these responses as the consequence of a single bacterial activity, the results suggest that the most effective strains combined several complementary functions that collectively improved plant resource acquisition and performance. This interpretation is consistent with the multifunctional nature of PGPR, in which hormonal modulation, nutrient mobilization, root development, and stress mitigation can interact to produce a plant response greater than that expected from any individual trait [47,48,49,50,51,52,53]. The ability of selected strains to improve both productivity and IWUE under DI is particularly relevant because it indicates that microbial inoculation may help reduce the trade-off between water saving and yield preservation.

The HCA and PCA consistently classified the 51 indigenous *Bacillus* strains into four functionally distinct groups according to their PGP characteristics and their effects on wheat growth, productivity, and IWUE (Figure 4 and Figure 5). Rather than simply confirming differences among strains, the multivariate analyses provided an integrated view of the relationships between bacterial functional traits and plant performance. Both analyses clearly identified Group 1, comprising *Bacillus cereus A2*, *B. pumilus D2* and E3, and *B. safensis D5*, as the elite cluster. These strains consistently produced the highest values for TLA, TFW, TDW, SpN, SpW, SN, GY, BY, and IWUE under both irrigation regimes, while also exhibiting the greatest PGP potential through the highest IAA and NH_3_ production (Table 5). Their position in the PCA, closely associated with IAA, NH_3_, and productivity-related traits (Figure 5), indicates that superior agronomic performance was linked to a combination of functional attributes rather than to a single PGP characteristic. This provides direct support for the integrated screening strategy adopted in this study. In contrast, Group 2 represented the least effective cluster, exhibiting the lowest mean values for most growth and productivity-related traits and IWUE under both irrigation regimes. Although this group recorded the highest HI under FI and TN under DI, it was characterized by the lowest IAA production and only moderate NH_3_ production (Table 5), explaining its limited overall growth-promoting capacity. Group 3 ranked second for most productivity-related traits, including TLA, TDW, SN, GY, BY, and IWUE under both irrigation regimes, as well as SpN and SpW under DI (Table 5). Its intermediate position in the PCA biplot, close to productivity-related traits under DI, together with the second-highest IAA and NH_3_ production (Figure 5), indicates good drought adaptation and considerable potential for maintaining wheat productivity under water-limited conditions. Group 4, comprising 21 strains and the uninoculated control, exhibited a distinct functional profile (Figure 4), characterized by relatively strong effects on morphological traits but less consistent improvements in wheat productivity, particularly under DI conditions. The strains in this group were also located near PH, LN, TN, and HI on the positive side of PC2. This separation between morphological promotion and yield improvement further demonstrates that enhanced vegetative growth alone is not sufficient for identifying the most agronomically valuable bioinoculants. The close agreement between HCA and PCA therefore strengthens confidence in the classification and demonstrates the value of multivariate approaches for distinguishing strains according to their integrated effects on microbial traits and plant performance.

The superior performance of the elite indigenous strains identified in Groups 1 and 3 agrees well with previous studies demonstrating the multifunctional roles of *Bacillus* spp. in promoting plant growth and drought tolerance. The superior strains identified in the present study, including *B. cereus* A2, *B. pumilus* D2 and E3, *B. subtilis* S-1, S-7, and E4, *B. amyloliquefaciens* Inh10, *B. safensis* D5 and M1-1, *B. sonorensis* S3, *B. thuringiensis* S-6, and *B. crassostreae* SS3, have all been reported to enhance plant growth and stress tolerance through multiple complementary mechanisms. In particular, *B. cereus* has been reported to improve biomass accumulation, nutrient uptake, photosynthetic performance, and drought tolerance through IAA and siderophores production, ACC deaminase activity, enhanced antioxidant activity and improved osmotic regulation [25,64]. Likewise, *B. subtilis*, *B. amyloliquefaciens*, *B. pumilus*, and *B. safensis* have been shown to stimulate root development, nutrient acquisition, chlorophyll biosynthesis, and tolerance to abiotic stresses through the production of phytohormones, volatile organic compounds, ACC deaminase, and other bioactive metabolites [63,65,66,67]. Although fewer studies are available for *B. sonorensis* and *B. crassostreae*, both species have been recognized as promising PGPR because of their ability to produce growth-promoting metabolites and enhance plant performance under stressful conditions [68]. Similarly, *B. thuringiensis* has been reported to improve drought tolerance by increasing plant survival, photosynthetic activity, and biomass accumulation under water-deficit conditions [69]. Consistent with these findings, previous studies have reported significant improvements in wheat growth and grain yield following inoculation with *Bacillus* spp., including grain-yield increases of 19–42% under both optimal and deficit irrigation conditions [24,28,65,67]. The present results extend these observations by demonstrating that strains belonging to different *Bacillus* lineages can exhibit similarly high agronomic performance, whereas closely related strains may differ substantially in their effects on wheat. Thus, functional screening at the strain level is more informative than taxonomic identity alone for selecting effective bioinoculants. Therefore, the elite indigenous strains identified in the present study represent promising candidates for the development of locally adapted biofertilizers capable of enhancing wheat productivity and IWUE under both optimal and water-limited conditions in arid regions.

The superior performance of the elite indigenous strains identified in the present study agrees well with previous reports describing the multifunctional roles of *Bacillus* spp. in promoting plant growth and drought tolerance. Collectively, these findings validate the effectiveness of the multistage screening strategy employed in this study for identifying highly efficient indigenous PGPR and provide strong biological support for their use in improving wheat performance under water-limited conditions.

## 5. Conclusions

This study demonstrated substantial variation among indigenous *Bacillus* strains in their plant growth-promoting capacity and drought-mitigating potential. The integrated screening approach combining PGP characterization, greenhouse evaluation, and multivariate analyses successfully identified *Bacillus cereus* A2, *B. pumilus* D2 and E3, and *B. safensis* D5 as elite strains with superior and consistent effects on wheat growth, productivity, and IWUE under both full and deficit irrigation. Several additional strains, including *B. subtilis* S-1, S-7, and E4, *B. amyloliquefaciens* Inh10, *B. safensis* M1-1, *B. sonorensis* S3, *B. thuringiensis* S-6, and *B. crassostreae* SS3, also showed considerable potential for improving wheat performance. Overall, the findings highlight that indigenous *Bacillus* strains from arid agricultural environments represent a valuable reservoir of functionally diverse PGPR, and that strain-level integrated screening is more informative than selection based solely on individual PGP traits. Accordingly, the elite indigenous strains identified in this study provide promising candidates for further field validation and for the development of locally adapted bioinoculants aimed at sustaining wheat productivity and improving IWUE in arid and semi-arid agroecosystems.

Despite these promising findings, several limitations of the present study should be acknowledged. First, the evaluation was conducted over a single growing season under controlled greenhouse conditions, which may not fully reflect the environmental variability and complex dynamics of field agroecosystems. Therefore, the performance and stability of the elite *Bacillus* strains identified in this study require validation under field conditions across multiple growing seasons, soil types, multiple wheat cultivars with different genetic backgrounds, and environmental conditions. Second, although 16S rRNA gene sequencing provided appropriate taxonomic identification of the bacterial isolates, it does not directly confirm the presence, expression, or functional contribution of genes associated with specific PGP traits. Future studies should therefore incorporate genomic or targeted molecular analyses of PGP-related functional genes, together with physiological and biochemical measurements, to further clarify the biological basis of the superior performance of the elite strains. In addition, future research should evaluate the field efficacy and consistency of these elite strains across diverse soil types and environmental conditions, investigate their compatibility and effectiveness in consortium formulations, and optimize suitable liquid or solid carrier formulations to enhance their stability, delivery, and potential for commercial application in arid agriculture. Such validation would strengthen the practical relevance of the present findings and facilitate the development of reliable, locally adapted *Bacillus*-based bioinoculants for sustainable wheat.

## Figures and Tables

**Table 1:** Identification code, scientific name, GenBank accession number (Acc.), sequence similarity (%), and plant growth-promoting (PGP) traits, including indole-3-acetic acid (IAA, µg mL^−1^), ammonia (NH_3_, mg mL^−1^), potassium-solubilizing activity (K), and siderophore production (Sid), of the *Bacillus* strains evaluated in this study.

Code	Scientific Name	Acc.	Similarity	IAA	NH_3_	K	Sid
D2	*Bacillus pumilus* strain D2	*PX*375674	100.00%	159.96 ± 4.01 A	14.88 ± 0.08 B	−	−
Inh10	*Bacillus amyloliquefaciens* strain Inh10	*PX*375676	100.00%	53.76 ± 4.44 G	7.49 ± 0.07 K–N	−	+
M1-1	*Bacillus safensis* strain M1-1	*PX*375679	99.09%	32.63 ± 1.56 O–Q	8.17 ± 0.07 H–I	−	+++
A2	*Bacillus cereus* strain A2	*PZ*016799	97.45%	115.34 ± 1.48 B	16.19 ± 0.21 A	+	+
SS3	*Bacillus crassostreae* strain SS3	*PX*375696	99.65%	93.12 ± 1.59 C	1.84 ± 0.09 Y	−	−
B2	*Bacillus subtilis* strain B2	*PX*376447	98.23%	18.91 ± 0.82 X–Y	7.52 ± 0.11 J–N	−	+
B3	*Bacillus halotolerans* strain B3	*PX*376448	99.59%	19.65 ± 1.06 W–Y	6.75 ± 0.19 Q–R	−	+
B4	*Bacillus amyloliquefaciens* strain B4	*PX*376449	97.45%	38.8 ± 0.40 M–N	7.73 ± 0.17 J–L	−	+
B6	*Bacillus halotolerans* strain B6	*PX*376451	97.02%	46.31 ± 2.42 H–K	8.53 ± 0.16 G–H	−	+++
B7	*Bacillus subtilis* strain B7	*PX*376452	96.48%	48.45 ± 1.07 H–I	3.21 ± 0.15 W	−	++
B8	*Bacillus amyloliquefaciens* strain B8	*PX*376453	99.85%	29.31 ± 0.37 Q–U	7.42 ± 0.11 L–O	−	+
B9	*Bacillus amyloliquefaciens* strain B9	*PX*376454	98.99%	36.33 ± 2.14 N–O	7.82 ± 0.19 I–K	−	+++
B10	*Bacillus subtilis* strain B10	*PX*376455	97.24%	35.24 ± 3.86 N–O	9.36 ± 0.50 F	−	+++
B11	*Bacillus subtilis* strain B11	*PX*376456	99.82%	27.57 ± 2.48 S–U	7.88 ± 0.10 I–J	−	++
B14	*Bacillus halotolerans* strain B14	*PX*376458	100.00%	19.06 ± 1.42 X–Y	6.96 ± 0.10 P–Q	−	+++
B15	*Bacillus subtilis* strain B15	*PX*376459	98.87%	27.16 ± 3.01 T–U	5.83 ± 0.21 T	−	++
B18	*Bacillus amyloliquefaciens* strain B18	*PX*376461	100.00%	28.55 ± 1.05 R–U	7.16 ± 0.15 N–P	+	+
B19	*Bacillus subtilis* strain B19	*PX*376462	100.00%	30.62 ± 0.95 Q–T	7.56 ± 0.17 J–M	−	+++
B20	*Bacillus amyloliquefaciens* strain B21	*PX*376463	96.96%	30.58 ± 0.69 Q–T	8.75 ± 0.09 G	−	+
B24	*Bacillus amyloliquefaciens* strain B24	*PX*376466	99.15%	22.96 ± 3.94 V–W	10.49 ± 1.11 E	−	+
B25	*Bacillus inaquosorum* strain B25	*PX*376467	97.29%	20.69 ± 0.45 W–X	7.42 ± 0.10 L–O	−	+++
B26	*Bacillus subtilis* strain B26	*PX*376468	97.62%	31.09 ± 1.99 Q–S	6.14 ± 0.12 S–T	−	+
B27	*Bacillus amyloliquefaciens* strain B27	*PX*376469	97.36%	16.28 ± 0.95 Y–Z	10.83 ± 0.16 E	−	+
B28	*Bacillus tequilensis* strain B28	*PX*376470	97.62%	26.95 ± 1.82 T–U	7.84 ± 0.13 I–K	−	++
B29	*Bacillus subtilis* strain B29	*PX*376471	97.31%	29.75 ± 2.36 Q–U	8.11 ± 0.08 I–I	−	+++
S-1	*Bacillus subtilis* strain 1	*PX*376476	98.44%	49.2 ± 2.07 H–I	12.24 ± 0.24 D	−	++
S-3	*Bacillus sonorensis* strain 3	*PX*376477	98.11%	13.76 ± 0.26 Z–a	7.74 ± 0.07 J–L	−	++
S-4	*Bacillus subtilis* strain 4	*PX*376478	99.17%	35.11 ± 1.54 N–P	12.63 ± 0.20 C	−	++
S-6	*Bacillus thuringiensis* strain 6	*PX*376479	100.00%	82.8 ± 2.38 D	5.44 ± 0.12 U–U	−	+
S-7	*Bacillus subtilis* strain 7	*PX*376480	95.16%	18.17 ± 1.19 X–Y	12.89 ± 0.24 C	−	+++
S-12	*Bacillus rugosus* strain 12	*PX*376484	96.36%	31.48 ± 1.48 P–R	10.83 ± 0.11 E	+	++
S-14	*Bacillus subtilis* strain 14	*PX*376485	98.09%	26.32 ± 3.47 U–V	7.11 ± 0.04 O–P	−	+
D1	*Bacillus safensis* strain D1	*PX*376486	100.00%	41.08 ± 3.08 L–M	8.67 ± 0.05 G	−	+
D4	*Cytobacillus firmus* strain D4	*PX*376487	91.49%	44.68 ± 1.30 J–L	7.27 ± 0.12 M–P	−	+
D5	*Bacillus safensis* strain D5	*PX*376488	97.71%	43.2 ± 2.67 K–L	16 ± 0.16 A	−	+
E3	*Bacillus pumilus* strain E3	*PX*376490	93.63%	64.55 ± 5.29 F	11.94 ± 0.16 D	−	+
E4	*Bacillus subtilis* strain E4	*PX*376491	100.00%	73.54 ± 1.59 E	10.69 ± 0.17 E	−	+
E6	*Bacillus subtilis* strain E6	*PX*394465	98.04%	49.44 ± 1.14 H–I	9.35 ± 0.23 F	−	++
F1	*Bacillus stratosphericus* strain F1	*PZ*016803	100%	42.96 ± 1.16 K–L	7.26 ± 0.17 M–P	−	++
F2	*Bacillus amyloliquefaciens* strain F2	*PX*394466	100.00%	21.08 ± 1.40 W–X	7.86 ± 0.21 I–J	−	+
P12	*Bacillus altitudinis* strain P12	*PX*394486	98.96%	47.37 ± 1.07 H–J	6.56 ± 0.21 R	−	+
Inh1	*Bacillus subtilis* strain Inh1	*PX*394467	100.00%	41.42 ± 3.70 L–M	3.2 ± 0.03 W	−	−
Inh2	*Bacillus subtilis* strain Inh2	*PX*394468	96.89%	46.05 ± 2.50 I–K	2.24 ± 0.09 X	−	++
Inh3	*Bacillus subtilis* strain Inh3	*PX*394469	97.76%	42.91 ± 0.05 K–L	1.94 ± 0.08 X–Y	−	++
Inh5	*Bacillus subtilis* strain Inh5	*PX*394470	97.44%	49.13 ± 1.77 H–I	3.86 ± 0.16 V–V	−	++
Inh6-1	*Bacillus methylotrophicus* strain Inh6-1	*PX*394471	99.57%	46.31 ± 2.07 H–K	7.7 ± 0.10 J–L	−	+
Inh8	*Bacillus amyloliquefaciens* strain Inh8	*PX*394474	100.00%	49.84 ± 2.22 H	7.49 ± 0.09 K–N	−	+++
AB3	*Bacillus aryabhattai* strain AB3	*PX*394463	100.00%	49.42 ± 3.49 H–I	7.04 ± 0.14 P–Q	−	+++
AB4	*Bacillus cereus* strain AB4	*PX*394464	95.76%	29.26 ± 4.29 Q–U	6.48 ± 0.12 R–S	−	+
AB5	*Bacillus atrophaeus* strain AB5	*PZ*016800	93.33%	11.51 ± 0.40 ab	0.07 ± 0.03 Z	−	−
Ph4	*Bacillus aerophilus* strain Ph4	*PX*394489	100.00%	8.77 ± 2.12 b	0.01 ± 0.00 Z	−	+

Values are the means ± standard error of three replicates (n = 3). Means sharing at least one letter are not significantly different according to Tukey’s HSD test at *p* ≤ 0.05. Ranges represent all consecutive Tukey grouping letters within the range (e.g., C–H = C, D, E, F, G, H). For qualitative assays, “−” indicates no detectable activity, “+” indicates low activity, “++” indicates moderate activity, and “+++” indicates very high activity.

**Table 2:** Mean comparisons of different growth- and yield-related traits under full irrigation (FI) and deficit irrigation (DI), and the percentage decrease or increase in these traits under DI relative to FI.

Traits	FI	DI	Decreases or Increases (%)
Plant height (cm) (PH)	59.13 ± 6.38 a	51.37 ± 5.63 b	13.1
Tiller number per plant (TN)	4.50 ± 0.59 a	3.52 ± 0.42 a	21.8
Leaf number per plant (LN)	17.51 ± 2.79 a	11.13 ± 1.74 b	36.4
Total leaf area (cm^2^ plant^−1^) (TLA)	192.28 ± 27.50 a	74.39 ± 12.74 b	61.3
Total fresh weigh (g plant^−1^) (TFW)	11.78 ± 1.42 a	7.47 ± 1.14 b	36.6
Total dry weight (g plant^−1^) (TDW)	3.79 ± 0.45 a	2.21 ± 0.30 b	41.7
Spike number per plant (SpN)	4.78 ± 0.59 a	2.77 ± 0.30 b	42.1
Spike weight (g plant^−1^) (SpW)	3.95 ± 0.49 a	2.39 ± 0.37 b	39.5
Seed number per plant (SN)	69.54 ± 8.60 a	45.52 ± 7.88 b	34.5
Grain yield (kg m^−2^) (GY)	509.48 ± 58.0 a	345.49 ± 48.27 b	32.2
Biological yield (kg m^−2^) (BY)	1516.20 ± 200.2 a	911.59 ± 155.83 b	39.9
Harvest index (%) (HI)	33.83 ± 1.70 b	38.36 ± 2.31 a	13.4
Irrigation water use efficiency (kg m^−3^) (IWUE)	1.33 ± 0.15 b	1.85 ± 0.26 a	39.1

Data are presented as means ± standard error of three replicates (n = 3). The means that share the same lowercase letters within the same row indicate are not significantly different according to Tukey’s HSD test at *p* ≤ 0.05.

**Table 3:** Percentage change in different growth- and yield-related traits for each *Bacillus* strain relative to the uninoculated control under full irrigation conditions.

Strains	PH	TN	LN	TLA	TFW	TDW	SpN	SpW	SN	GY	BY	HI	IWUE
D2	0.3	8.1	−1.1	28.1	28.1	30.6	43.6	30.4	22.4	30.0	33.1	−0.5	30.0
Inh10	−0.2	−7.8	−6.9	7.6	7.3	3.0	0.0	7.6	12.9	1.9	6.8	−0.7	1.9
M1-1	4.5	−7.8	−9.6	15.0	10.4	12.0	7.3	11.7	8.1	6.5	12.5	−0.5	6.5
A2	1.8	−0.5	−0.6	38.0	28.1	36.7	27.3	46.8	40.8	36.9	49.7	−0.0	36.9
SS3	−0.0	−2.4	−7.6	23.0	23.6	25.4	−0.6	−0.0	32.5	20.0	30.6	−0.1	20.0
B2	−2.2	−9.7	−0.5	−8.9	−0.7	−5.9	−4.5	−0.8	−7.1	−8.3	−0.9	−0.5	−8.3
B3	−0.2	−4.3	−0.8	−0.6	14.8	−0.3	−0.6	0.7	−0.6	−0.3	−0.6	6.3	−0.3
B4	−0.3	−2.4	−7.6	−3.3	−0.6	−0.0	−0.8	−0.5	−0.2	−0.1	−1.0	0.6	−0.1
B6	−0.3	−9.7	−4.1	14.1	8.0	12.9	−0.6	9.2	13.2	7.9	8.2	0.2	7.9
B7	−0.2	−1.6	−4.8	−2.7	−0.3	−0.0	−0.5	−0.1	−0.2	−0.7	−0.4	−0.6	−0.7
B8	−0.9	−4.3	−0.8	−0.9	11.3	−0.1	−0.3	−0.1	−0.0	−0.1	−0.5	2.5	−0.1
B9	−0.9	−9.7	−0.4	−0.2	5.0	−0.7	10.9	6.3	−0.1	−0.3	−0.6	5.7	−0.3
B10	−0.3	−8.9	−0.3	11.5	15.7	6.8	−0.3	−0.2	19.0	8.3	13.6	−0.7	8.3
B11	−4.8	−7.0	−0.6	−1.0	−0.0	−0.6	−0.5	−0.3	−0.8	−3.7	−0.0	−0.0	−3.7
B14	−0.8	−9.7	−0.4	−5.1	6.0	−0.1	3.6	−0.8	−0.0	−0.4	−0.5	0.2	−0.4
B15	−0.4	−2.4	−3.9	8.3	5.2	6.7	10.9	4.5	11.9	1.8	3.6	−0.0	1.8
B18	−0.7	−4.3	5.6	−0.6	16.2	−0.5	3.6	5.5	−0.6	−0.4	−0.5	1.0	−0.4
B19	2.1	−5.1	−0.4	−0.6	5.2	−0.0	−0.3	−0.3	−0.5	−0.9	−0.2	7.1	−0.9
B20	2.7	−2.4	−1.3	8.8	4.4	9.0	3.6	7.1	7.0	8.7	9.4	−0.3	8.7
B24	−0.5	−1.6	−0.4	−0.5	7.7	−0.2	−4.5	4.1	1.0	−0.3	−0.6	0.1	−0.3
B25	2.4	−8.9	−0.9	−0.1	11.8	−0.5	−6.4	7.5	−0.7	−0.4	−0.6	−0.7	−0.4
B26	0.0	−3.5	−0.3	9.6	23.9	11.5	−0.1	10.1	5.2	3.1	6.9	−0.2	3.1
B27	0.9	−5.1	−5.7	9.3	10.2	10.7	−0.1	5.5	3.8	5.5	8.4	−0.4	5.5
B28	−0.6	−1.6	−1.3	9.5	15.6	8.2	−0.3	12.5	14.1	7.3	6.6	−0.8	7.3
B29	7.7	−7.0	−4.8	−3.5	1.9	−0.6	−0.3	5.9	−0.3	−0.6	−0.6	1.3	−0.6
S-1	−0.6	−2.4	−1.1	9.4	16.5	5.9	−0.8	14.0	13.9	3.1	10.2	−0.5	3.1
S-3	−0.2	−2.4	−5.7	17.2	20.3	18.9	5.5	18.5	20.2	17.0	17.6	−0.3	17.0
S-4	1.8	−3.5	3.7	1.4	6.5	−0.9	0.0	5.5	−0.6	−0.2	−0.8	−0.3	−0.2
S-6	6.8	−7.0	−3.0	13.8	19.4	15.5	1.8	5.3	8.8	4.5	9.1	−0.3	4.5
S-7	10.7	−2.4	−0.7	14.6	15.5	12.8	−0.5	11.0	16.1	5.4	15.3	−0.2	5.4
S-12	2.4	−0.8	25.9	27.8	27.1	11.5	12.7	14.5	17.8	6.5	18.8	−0.4	6.5
S-14	0.0	−2.4	16.7	2.0	−0.1.0	−0.5.0	5.5	6.2	−0.3.0	−0.5	−0.0	−0.1	−0.5
D1	4.7	−1.6	16.7	18.8	22.1	5.7	−0.3	11.3	13.8	1.6	11.8	−0.2	1.6
D4	4.5	−6.2	17.6	7.6	10.2	6.6	14.5	9.6	8.7	6.7	13.2	−0.9	6.7
D5	7.1	−2.4	−0.8	37.8	46.4	33.3	38.2	50.2	34.8	29.6	39.1	−0.8	29.6
E3	10.7	−0.5	−0.6	32.0	25.2	35.6	38.2	52.0	35.2	34.5	31.6	1.0	34.5
E4	9.8	−7.8	−0.5	12.8	26.7	7.2	14.5	8.1	12.4	7.2	16.4	−0.6	7.2
E6	20.5	−8.9	29.6	−0.4	20.1	−0.2	−0.6	9.9	−0.0	−0.7	−0.6	−0.0	−0.7
F1	15.1	−5.1	−0.8	−1.8	2.2	−0.7	5.5	14.2	−0.2	−0.0	−0.9	1.4	−0.0
F2	17.2	−2.4	−3.9	−0.6	−0.5	−0.9	7.3	18.4	−0.5	−0.7	−0.9	−0.1	−0.7
P12	24.3	−2.4	22.2	9.9	17.7	5.9	12.7	23.5	11.0	6.1	12.4	−0.7	6.1
Inh1	12.8	−4.3	19.4	14.5	36.2	17.5	2.0	30.0	12.5	8.2	7.7	0.7	8.2
Inh2	8.9	−5.1	13.0	5.7	36.7	8.3	27.3	27.2	6.3	1.2	15.5	−1.7	1.2
Inh3	21.4	−2.4	4.6	7.9	18.4	10.9	14.5	17.1	11.5	3.5	8.5	−0.4	3.5
Inh5	20.8	−9.7	22.2	24.5	42.5	25.5	9.1	18.1	24.7	17.9	29.2	−0.1	17.9
Inh6-1	16.3	−7.0	20.4	−7.5	−0.3	−3.3	7.3	0.2	−0.6	−6.1	−0.8	−0.5	−6.1
Inh8	13.4	−4.3	14.8	11.4	32.2	13.6	12.7	37.2	19.3	11.2	15.6	−0.3	11.2
AB3	25.5	−0.5	3.7	−0.2	20.0	−0.2	9.2	19.0	−0.4	−0.3	−0.9	7.5	−0.3
AB4	24.0	−7.0	0.0	7.7	29.6	3.5	7.3	14.4	8.6	7.7	2.2	5.6	7.7
AB5	39.8	−1.6	25.0	8.3	53.7	7.1	7.3	9.7	9.2	7.5	3.8	3.2	7.5
Ph4	27.9	−7.0	8.3	−9.3	14.2	−0.7	5.5	18.3	−2.3	−0.9	−0.9	−0.7	−0.9

The color gradient indicates the magnitude and direction of the percentage change relative to the uninoculated control, ranging from dark red to dark green. Dark red represents the highest percentage reductions, whereas dark green represents the highest percentage increases; intermediate colors indicate intermediate value. The full names of *Bacillus* strain and trait abbreviations are presented in Table 1 and Table 2, respectively.

**Table 4:** Percentage change in different growth- and yield-related traits for each *Bacillus* strain relative to the uninoculated control under deficit irrigation conditions.

Strains	PH	TN	LN	TLA	TFW	TDW	SpN	SpW	SN	GY	BY	HI	IWUE
D2	11.2	−9.5	7.0	31.3	19.9	30.1	15.2	−0.8	52.5	24.9	33.4	−5.7	24.9
Inh10	7.2	4.8	12.3	10.7	3.7	6.7	6.1	6.8	16.4	7.3	5.5	4.5	7.3
M1-1	12.3	−4.8	−5.3	5.2	−4.5	9.2	−6.1	−3.5	12.4	1.9	10.1	−5.6	1.9
A2	8.7	0.0	−3.5	45.2	17.4	35.3	27.3	25.5	67.2	44.7	78.5	−19.7	44.7
SS3	−0.4	−4.8	8.8	16.6	3.8	16.4	27.3	23.0	33.8	18.9	15.2	3.9	18.9
B2	1.8	4.8	10.5	−5.6	−1.1	−2.7	6.1	−3.8	−0.9	−1.0	−5.3	4.6	−1.0
B3	10.5	19.0	49.1	−6.6	−9.8	−4.7	3.0	−1.5	−0.8	−1.6	−2.2	0.4	−1.6
B4	3.6	4.8	26.3	−6.7	−0.7	−3.6	9.1	−4.7	−0.8	−4.5	−7.1	2.3	−4.5
B6	−1.1	−9.5	5.3	−0.1	−4.9	−2.5	−0.0	−8.0	−0.1	−6.1	−4.0	−0.3	−6.1
B7	10.5	4.8	−5.3	−1.6	−2.1	−0.7	−0.1	−7.4	−0.1	−6.2	−4.5	−0.6	−6.2
B8	4.7	19.0	42.1	−3.3	−1.9	−3.1	15.2	−0.3	−0.7	−0.2	−0.1	1.9	−0.2
B9	5.8	9.5	14.0	−18.4	−8.3	−0.3	3.0	−0.8	−0.9	−0.1	−0.2	−0.2	−0.1
B10	6.2	4.8	42.1	−3.6	−2.0	−1.9	−0.0	−0.9	−0.9	−1.5	−0.0	−0.0	−1.5
B11	−2.9	19.0	−5.3	−9.9	−4.7	−0.5	3.0	−0.5	−0.1	−0.0	−0.9	1.7	−0.0
B14	−2.9	23.8	−22.8	−26.1	−0.0	−2.3	3.0	−7.5	−2.6	−8.4	−1.8	−5.1	−8.4
B15	−2.9	4.8	17.5	−4.7	−4.2	−1.6	3.0	−6.3	−0.9	−4.5	−0.7	−0.5	−4.5
B18	0.0	33.3	3.5	9.2	6.6	14.7	0.0	−0.4	20.3	6.7	4.2	3.2	6.7
B19	14.1	4.8	14.0	−2.7	−0.1	−2.1	0.0	−0.1	−1.0	−1.4	−0.0	−0.1	−1.4
B20	−5.8	−14.3	−12.3	−8.3	−0.6	−0.5	0.0	−4.8	−0.7	−0.1	−1.7	5.9	−0.1
B24	2.2	19.0	28.1	−15.8	−2.8	−2.8	−0.1	−0.5	−3.5	−2.0	−7.0	8.7	−2.0
B25	4.7	19.0	36.8	12.7	−0.2	7.2	−0.1	−9.2	11.0	1.6	8.6	−0.9	1.6
B26	19.9	−0.8	8.8	−4.2	−2.1	−2.0	−5.2	−6.5	−8.0	−1.9	−6.2	−0.2	−1.9
B27	4.3	0.0	7.0	−3.2	−3.1	−9.3	0.0	−0.7	−1.8	−0.1	−5.2	−0.0	−0.1
B28	5.4	−0.5	1.8	−1.9	−0.6	−0.7	−8.2	−0.5	−0.7	−0.9	−3.5	10.1	−0.9
B29	2.2	−0.5	3.5	−2.2	−0.4	−4.2	−0.0	−0.9	−0.9	−0.2	−0.9	−0.5	−0.2
S-1	9.4	−0.8	28.1	10.2	8.3	11.2	−5.2	−2.8	12.9	1.0	3.3	−0.9	1.0
S-3	−0.9	−0.8	8.8	14.8	0.9	9.8	3.0	−0.0	16.0	3.9	4.6	−0.1	3.9
S-4	10.1	14.3	40.4	14.1	11.8	10.1	6.1	−0.4	24.7	8.3	15.6	−0.7	8.3
S-6	6.2	−4.3	3.5	28.8	16.5	22.5	15.2	26.0	35.9	17.7	19.8	−0.2	17.7
S-7	8.0	−0.5	−0.5	11.3	3.1	6.1	3.0	5.2	17.2	10.2	10.0	0.7	10.2
S-12	1.4	0.0	42.1	−1.2	0.7	−0.7	18.2	−4.7	−0.9	−0.9	−0.3	−0.8	−0.9
S-14	0.7	−0.5	−7.5	18.5	9.8	7.1	3.0	−0.1	19.9	8.6	9.1	−0.3	8.6
D1	10.1	19.0	47.4	17.9	23.0	15.6	12.1	1.6	23.7	10.1	17.6	−0.0	10.1
D4	5.4	0.0	26.3	20.7	13.8	18.4	27.3	35.0	21.2	14.7	15.6	−0.9	14.7
D5	5.1	9.5	3.5	12.7	3.8	3.7	0.0	−2.0	8.7	7.0	18.6	−0.1	7.0
E3	10.1	−0.8	10.5	8.6	9.5	8.7	3.0	−3.5	11.6	1.9	3.5	−0.3	1.9
E4	22.8	−0.8	33.3	13.7	14.5	12.7	3.0	−0.4	22.2	11.0	13.3	−0.1	11.0
E6	36.6	9.5	33.3	14.5	13.7	12.9	6.1	8.7	21.8	8.7	7.8	2.3	8.7
F1	25.4	4.8	42.1	−0.3	1.7	−0.6	−2.1	−0.1	−0.9	−0.5	−0.3	1.7	−0.5
F2	31.5	4.8	26.3	20.5	18.0	17.4	−0.1	−0.9	9.5	6.4	10.7	−0.8	6.4
P12	25.0	4.8	45.6	−8.6	12.5	−0.8	−5.2	−0.7	−0.9	−0.2	−3.7	8.2	−0.2
Inh1	26.8	−4.3	−0.8	−6.4	5.8	−0.8	3.0	15.9	−0.9	−0.6	−5.2	12.7	−0.6
Inh2	33.0	−0.8	42.1	14.4	24.2	12.6	−0.0	13.7	15.3	5.2	10.8	−0.3	5.2
Inh3	23.9	−0.5	26.3	−0.1	9.1	−0.3	−0.0	16.2	−0.2	−0.1	−0.4	1.0	−0.1
Inh5	27.9	−0.8	35.1	18.1	39.1	12.8	0.0	13.6	20.3	7.2	21.0	−0.0	7.2
Inh6-1	22.5	−0.8	28.1	7.9	31.4	4.2	−0.1	4.7	8.9	0.6	5.0	−0.3	0.6
Inh8	12.0	−9.0	15.8	−3.5	8.7	−0.1	−0.1	9.1	−0.6	−0.6	−0.0	5.1	−0.6
AB3	30.1	−9.0	12.3	−0.0	3.5	−0.3	0.0	9.8	−0.8	−0.4	−3.2	3.3	−0.4
AB4	37.3	−3.8	15.8	−4.2	7.2	−0.0	−5.2	9.9	−0.9	−0.2	−1.4	2.8	−0.2
AB5	29.0	0.0	35.1	9.3	20.6	3.4	−5.2	2.6	15.4	3.0	10.2	−0.9	3.0
Ph4	40.2	−4.3	26.3	−0.4	6.0	−0.1	−3.3	15.5	−0.4	−0.4	−0.4	−0.3	−0.4

The color gradient indicates the magnitude and direction of the percentage change relative to the uninoculated control, ranging from dark red to dark green. Dark red represents the highest percentage reductions, whereas dark green represents the highest percentage increases; intermediate colors indicate intermediate value. The full names of *Bacillus* strain and trait abbreviations are presented in Table 1 and Table 2, respectively.

**Table 5:** Mean values of growth- and yield-related traits under full and deficit irrigation, together with plant growth-promoting (PGP) traits, for each *Bacillus* strains group identified by hierarchical cluster analysis based on the heatmap of all traits measured under both irrigation treatments and PGP traits. Trait abbreviations are defined in the caption of Figure 3.

Plant Traits	Full Irrigation				Deficit Irrigation			
	Group-1	Group-2	Group-3	Group-4	Group-1	Group-2	Group-3	Group-4
PH	58.96	54.23	57.06	63.92	50.04	47.94	48.88	55.33
TN	4.54	4.50	4.27	4.57	3.46	3.68	3.40	3.45
LN	15.79	15.80	15.44	19.97	9.92	10.69	10.60	11.89
TLA	245.79	176.91	206.84	189.82	92.85	61.98	85.04	77.32
TFW	13.50	10.90	11.76	12.20	8.29	6.18	7.76	8.26
TDW	4.80	3.61	3.98	3.69	2.63	1.91	2.46	2.29
SpN	6.27	4.39	4.60	4.90	3.06	2.74	2.89	2.70
SpW	5.13	3.60	3.84	4.06	2.40	2.09	2.57	2.56
SN	86.85	65.29	74.04	68.23	58.27	38.53	52.30	46.46
GY	654.02	484.19	524.24	498.53	419.58	298.46	381.17	357.52
BY	1973.47	1407.24	1597.92	1492.48	1207.87	782.02	999.80	931.66
HI	33.12	34.53	32.84	33.73	35.49	38.46	38.41	38.78
IWUE	1.71	1.27	1.37	1.30	2.25	1.60	2.05	1.92
Plant Growth-Promoting traits								
	Group-1		Group-2		Group-3		Group-4	
IAA (µg mL^−1^)	95.76		29.72		47.32		36.45	
NH_3_ (mg mL^−1^)	14.75		7.71		8.55		5.92	

## Data Availability

All data are presented within the article and Supplementary Materials. The gene sequences of the *Bacillus* strains have been deposited in the NCBI GenBank database under the accession numbers provided in Table 1.

## Supplementary Materials

The following supporting information can be downloaded at https://www.mdpi.com/article/10.3390/life16091511/s1: Table S1: Results of analysis of variance (Mean square values) for the effects of irrigation (I), *Bacillus* strains (BS), and their interactions on vegetative growth measured at 85 days after sowing, yield, yield components, and irrigation water use efficiency of wheat; Table S2: Mean comparison of plant height (PH), tiller number (TN), leaf number (LN), and total leaf area (TLA) of wheat inoculated with different *Bacillus* strains (BS) under full irrigation (FI) and deficit irrigation (DI); Table S3: Mean comparison of total fresh weight (TFW), total dry weight (TDW), spike number (SpN), spike weight (SpW), and seed number (SN) of wheat inoculated with different *Bacillus* strains (BS) under full irrigation (FI) and deficit irrigation (DI); Table S4: Mean comparison of grain yield (GY), biological yield (BY), harvest index (HI), and irrigation water use efficiency (IWUE) of wheat inoculated with different *Bacillus* strains (BS) under full irrigation (FI) and deficit irrigation (DI), Table S5: Eigenvalue, cumulative variability, and factor loadings of the first two principal components (PCs) for different growth- and yield-related traits and plant growth-promoting traits under full irrigation (FI) and deficit irrigation (DI).
