# Supplementary material for: Integrated Screening Identifies Elite Indigenous Bacillus Strains for Enhancing Wheat Productivity and Irrigation Water-Use Efficiency Under Full and Deficit Irrigation
Source: Life (Basel). 2026 Sep 10;16(9):1511. doi: 10.3390/life16091511 (PMC13608201; doi:10.3390/life16091511)
Supplement: Supplementary file 1 [file life-16-01511-s001.zip › life-4471177-supplementary.pdf]

Table S1. Results of analysis of variance (Mean square values) for the effects of irrigation (I), Bacillus strains (BS), and their interactions on vegetative growth measured at 85 days after sowing, yield, yield components, and irrigation water use efficiency of wheat

S.O.V.	DF	PH	TN	LN	TLA	TFW	TDW
Rep.	2	175.17	2.25	70.49	4915.0	55.59	4.01
I	1	4696.51**	74.54ns	3176.35**	1083900.0***	1452.56**	195.05**
Error 1	2	82.11	4.39	16.73	418.0	27.54	1.41
BS	51	196.11***	0.89*	23.3***	1842.0***	7.36***	0.60***
I × BS	51	21.05*	0.69*	9.11*	913.0***	2.57*	0.27*
Error 2	204	33.84	0.97	7.78	553.0	2.72	0.276
CV (%)		10.53	24.60	19.48	17.64	17.14	17.49

S.O.V.	DF	SpN	SpW	SN	GY	BY	HI	IWUE
Rep.	2	1.47	5.57	2086.70	151247.0	867816	20.00	2.05
I	1	316.30***	190.16**	44977.50***	2097790.0**	2851000**	1604.26**	21.20**
Error 1	2	0.038	3.955	12.3	8550.0	145388	17.03	0.0515
BS	51	0.77***	0.71***	301.50***	12596.0***	155952.0***	14.99*	0.19***
I × BS	51	0.56**	0.42**	107.00*	4485.0*	37186.20*	9.66*	0.08*
Error 2	204	0.389	0.255	108.8	5592.0	31201.6	31.21	0.0769
CV (%)		16.51	15.93	18.13	17.49	14.55	15.48	17.40

DF indicates degrees of freedom. *, **, and *** indicate significance at the 0.05, 0.01, and 0.001 probability levels, respectively, while NS indicates non-significant differences. PH, TN, LN, TLA, TFW, TDW, SpN, SpW, SN, GY, BY, HI, and IWUE denote plant height (cm), tiller number per plant, leaf number per plant, total leaf area (cm^2 per plant), total fresh weight (g per plant), total dry weight (g per plant), spike number per plant, spike weight (g per plant), seed number per plant, grain yield (kg m^{-2}), biological yield (kg m^{-2}), harvest index (%), and irrigation water use efficiency (kg m^{-3}), respectively.

Table S2. Mean comparison of plant height (PH), tiller number (TN), leaf number (LN), and total leaf area (TLA) of wheat inoculated with different *Bacillus st*rains (BS) under full irrigation (FI) and deficit irrigation (DI).

BS	PH (cm)		TN		LN		TLA (cm^2 plant^{-1})	
	FI	DI	FI	DI	FI	DI	FI	DI
Control	56.17 i-C	46.00 F-J	6.17 ab	3.50 f-k	18.00 c-n	9.50 C-I	183.45 f-r	74.62 u-y
D2	56.33 i-B	51.17 q-J	6.67 a	3.17 h-k	16.00 i-u	10.17 A-I	234.97a-d	98.01 u-v
Inh10	55.5 j-E	49.33 v-J	3.83 d-k	3.67 e-k	13.17 o-F	10.67 y-I	197.33d-n	82.62 u-y
M1-1	58.67 f-v	51.67 q-J	3.83 d-k	3.33 g-k	12.67 r-G	9.00 F-I	210.93c-g	78.53 u-y
A2	57.17 h-z	50 t-J	3.67 e-k	3.5 f-k	12.50 s-G	9.17 E-I	253.23 a	108.31t-u
SS3	53.33 n-I	45.83 G-J	4.17 c-k	3.33 g-k	14.83 k-z	10.33 z-I	225.67a-e	86.99 u-y
B2	49.33 v-J	46.83 C-J	4.33 c-j	3.67 e-k	16.83 g-s	10.5 z-I	148.71q-s	63 v-y
B3	53.83 m-I	50.83 r-J	4.67 b-h	4.17 c-k	17.50 d-p	14.17m-B	167.68 j-s	62.24 v-y
B4	51.5 q-J	47.67 A-J	4.17 c-k	3.67 e-k	14.83 k-z	12.00u-H	140.71 s-t	62.17 v-y
B6	54.33 l-H	45.5 H-J	4.33 c-j	3.17 h-k	13.67 n-E	10.00 A-I	209.38c-g	52.16 y-y
B7	52.67 o-J	50.83 r-J	4.83 b-g	3.67 e-k	15.33 j-x	9.00 F-I	160.16n-s	65.97 v-y
B8	51.17 q-J	48.17 x-J	4.67 b-h	4.17 c-k	17.5 d-p	13.5 n-F	169.03 j-s	64.69 v-y
B9	52.83 n-I	48.67 w-J	4.33 c-j	3.83 d-k	16.67 g-t	10.83 x-I	166.55k-s	60.92 v-y
B10	53.17 n-I	48.83 w-J	5 b-f	3.67 e-k	16.50 h-u	13.5 n-F	204.51 c-j	64.49 v-y
B11	47.83 z-J	44.67 I-J	4.5 c-i	4.17 c-k	17.00 f-s	9.00 F-I	163.25 l-s	59.76w-y
B14	55.17 k-G	44.67 I-J	4.33 c-j	4.33 c-j	14.33 l-A	7.33 I-I	155.74p-s	55.13 x-y
B15	54.83 l-H	44.67 I-J	4.17 c-k	3.67 e-k	15.5 j-w	11.17 v-I	198.71d-m	63.68 v-y
B18	51.83 p-J	46 F-J	4.67 b-h	4.67 b-h	19.00 a-k	9.83 A-I	171.31h-s	81.49 u-y
B19	57.33 h-y	52.5 o-J	4 c-k	3.67 e-k	16.67 g-t	10.83 x-I	169.45 i-s	65.12 v-y
B20	57.67h-w	43.33 J-J	4.17 c-k	3 i-k	14.17m-B	8.33 G-I	199.51d-m	60.95 v-y
B24	55.33 j-F	47 B-J	4.83 b-g	4.17 c-k	16.67 g-t	12.17 t-H	169.63 i-s	62.83 v-y
B25	57.5 h-x	48.17 x-J	5 b-f	4.17 c-k	17.67 d-o	13.00 p-F	179.54g-r	84.08 u-y
B26	56.17 i-C	55.17 k-G	5.33 a-d	3.33 g-k	16.5 h-u	10.33 z-I	201.1 d-k	64.01 v-y
B27	56.67 h-A	48 y-J	4 c-k	3.5 f-k	15.17 j-y	10.17 A-I	200.54d-l	57.32 x-y
B28	55.83 j-D	48.5 w-J	4.83 b-g	3.17 h-k	14.17m-B	9.67 B-I	200.96d-l	65.7 v-y
B29	60.5 d-q	47 B-J	4.5 c-i	3.17 h-k	15.33 j-x	9.83 A-I	158.73o-s	65.52 v-y
S-1	55.83 j-D	50.33 t-J	4.17 c-k	3.33 g-k	16.00 i-u	12.17 t-H	200.67d-l	82.21 u-y
S-3	53.83 m-I	44.67 I-J	4.17 c-k	3.33 g-k	15.17 j-y	10.33 z-I	215.05b-g	85.65 u-y
S-4	57.17 h-z	50.67 s-J	5.33 a-d	4 c-k	18.67b-m	13.33 o-F	185.96f-q	85.12 u-y
S-6	60 e-s	48.83 w-J	4.5 c-i	3 i-k	15.67 j-v	9.83 A-I	208.77c-h	96.14u-w
S-7	62.17 c-n	49.67 u-J	4.17 c-k	3.17 h-k	17.33 e-q	9.17 E-I	210.32c-g	83.08 u-y
S-12	57.5 h-x	46.67 D-J	5.5 a-c	3.5 f-k	22.67 ab	13.5 n-F	234.44a-d	66.28 v-y
S-14	56.17 i-C	46.33 E-J	4.17 c-k	3.17 h-k	21.00 a-h	7.83 H-I	187.11f-p	88.42 u-y
D1	58.83 e-u	50.67 s-J	4.83 b-g	4.17 c-k	21.00 a-h	14.00 n-C	217.88 a-f	87.96 u-y
D4	58.67 f-v	48.5 w-J	5.17 a-e	3.5 f-k	21.17 a-g	12.00u-H	197.38d-n	90.09 u-x
D5	60.17 e-r	48.33 w-J	4.17 c-k	3.83 d-k	17.5 d-p	9.83 A-I	252.81 a	84.06 u-y
E3	62.17 c-n	50.67 s-J	3.67 e-k	3.33 g-k	17.17 e-r	10.50 z-I	242.16a-c	81.03 u-y
E4	61.67 c-o	56.5 h-A	3.83 d-k	3.33 g-k	16.83 g-s	12.67 r-G	206.89 c-i	84.81 u-y
E6	67.67 b-g	62.83b-m	5 b-f	3.83 d-k	23.33 a	12.67 r-G	169.95 i-s	85.42 u-y
F1	64.67 b-j	57.67h-w	4 c-k	3.67 e-k	17.50 d-p	13.50 n-F	161.88 m-s	70.63 u-y
F2	65.83 b-h	60.5 d-q	4.17 c-k	3.67 e-k	15.50 j-w	12.00u-H	169.56 i-s	89.94 u-y
P12	69.83 a-d	57.5 h-x	4.17 c-k	3.67 e-k	22 .00a-d	13.83 n-D	201.62 d-k	60.74 v-y
Inh1	63.33 b-l	58.33 g-v	4.67 b-h	3 i-k	21.50 a-f	9.33 D-I	210.12 c-g	62.36 v-y
Inh2	61.17 c-p	61.17 c-p	4 c-k	3.33 g-k	20.33 a-i	13.50 n-F	193.82 e-o	85.36 u-y
Inh3	68.17 b-e	57 h-A	4.17 c-k	3.17 h-k	18.83 a-l	12.00u-H	197.96 d-n	68.54 v-y

Inh5	67.83 b-f	58.83 e-u	4.33 c-j	3.33 g-k	22.00 a-d	12.83 q-G	228.44 a-e	88.13 u-y
Inh6-1	65.33 b-i	56.33 i-B	4.5 c-i	3.33 g-k	21.67 a-e	12.17 t-H	151.41 p-s	80.53 u-y
Inh8	63.67 b-l	51.5 q-J	4.67 b-h	2.83 j-k	20.67 a-h	11.00 w-I	204.45 c-j	64.55 v-y
AB3	70.5 a-c	59.83 e-s	3.67 e-k	2.83 j-k	18.67b-m	10.67 y-I	164.79k-s	69.38 v-y
AB4	69.67 a-d	63.17b-m	4.5 c-i	2.67 k	18 c-n	11 w-I	197.55d-n	64.03 v-y
AB5	78.5 a	59.33 e-t	4.83 b-g	3.5 f-k	22.5 a-c	12.83 q-G	198.61d-m	81.58 u-y
Ph4	71.83 ab	64.5 b-k	4.5 c-i	3 i-k	19.5 a-j	12 u-H	147.98 r-s	72.09 u-y
**Mean**	**59.13A**	**51.37B**	**4.50A**	**3.52A**	**17.51A**	**11.13B**	**192.28A**	**74.39B**

Values are the means of three replicates. Means sharing at least one letter are not significantly different according to Tukey's HSD test at P ≤ 0.05. Hyphenated letter ranges represent all consecutive Tukey grouping letters within the range (e.g., C–H = C, D, E, F, G, H).

Table S3. Mean comparison of total fresh weight (TFW), total dry weight (TDW), spike number (SpN), spike weight (SpW), and seed number (SN) of wheat inoculated with different *Bacillus st*rains (BS) under full irrigation (FI) and deficit irrigation (DI).

BS	TFW (g plant^{-1})		TDW (g plant^{-1})		SpN		SpW (g plant^{-1})		SN	
	FI	DI	FI	DI	FI	DI	FI	DI	FI	DI
Control	10.23 h-x	7.36 y-J	3.58 f-m	2.2 x-D	4.58 c-g	2.75 l-n	3.54 h-t	2.46 w-H	65.17 e-t	43.17 x-F
D2	13.11 a-g	8.83 o-E	4.68 a-d	2.87 m-z	6.58 a	3.17 j-n	4.62 a-e	2.2 C-I	79.75 a-f	65.83 e-s
Inh10	10.98 e-s	7.63 x-J	3.69 e-m	2.35 v-D	4.58 c-g	2.92 k-n	3.82 e-p	2.63 u-H	73.58 b-m	50.25 s-E
M1-1	11.29 d-q	7.03 z-J	4.01 b-j	2.41 v-D	4.92 b-e	2.58 l-n	3.96 e-n	2.38 z-I	70.42 d-n	48.5 t-F
A2	13.1 a-g	8.64 p-G	4.9 a	2.98 l-y	5.83 ab	3.5 h-l	5.2 a-c	3.09 o-B	91.75 a	72.17b-m
SS3	12.65 b-i	7.64 x-J	4.49 a-e	2.57 p-C	4.42 c-h	3.5 h-l	3.3 l-y	3.03 p-C	86.33 a-d	57.75 l-A
B2	9.44 l-B	5.81 H-J	3.01 l-x	1.92 A-D	3.92 f-j	2.92 k-n	3.3 l-y	2.12 D-I	54 n-C	39.75 C-F
B3	11.74 d-n	5.9 G-J	3.5 f-n	1.88 A-D	4.42 c-h	2.83 l-n	3.57 h-r	1.93 F-I	63.48 f-w	34.18 E-F
B4	9.66 k-A	5.1 J	3.48 f-n	1.69 D	4.5 c-g	3 j-n	3.42 i-u	2.1 D-I	58.5 l-z	40.25 C-F
B6	11.05 e-r	6.27 E-J	4.05 a-j	1.71 C-D	4.42 c-h	2.67 l-n	3.87 e-p	1.77 H-I	73.75 b-m	34.5 E-F
B7	10 i-y	6.47 C-J	3.44 f-o	1.99 A-D	4.33 c-h	2.58 l-n	3.33 j-w	2.03 D-I	60.5 j-w	41.42 A-F
B8	11.39 d-p	5.75 I-J	3.37 g-r	1.92 A-D	4.25 d-h	3.17 j-n	3.29 l-y	2.41 z-I	61.25 h-w	39.83 C-F
B9	10.74 f-u	6.01 F-J	3.52 f-n	1.98 A-D	5.08 b-e	2.83 l-n	3.77 e-p	2.42 z-H	61.83 g-w	40.17 C-F
B10	11.84c-m	5.74 I-J	3.83 d-l	1.94 A-D	4.25 d-h	2.67 l-n	3.54 h-s	2.37 z-I	77.52 a-i	39.75 C-F
B11	10.03 i-y	6.28 E-J	3.28 i-u	2.04 z-D	4.33 c-h	2.83 l-n	3.36 i-v	1.96 F-I	59.42 k-x	40.08 C-F
B14	10.85 e-t	6.77 B-J	3.44 f-o	1.71 C-D	4.75 c-g	2.83 l-n	3.23 m-z	1.54 I-I	61.25 h-w	33.42 F
B15	10.76 e-u	6.32 D-J	3.82 d-l	1.95 A-D	5.08 b-e	2.83 l-n	3.7 g-q	1.82 G-I	72.92 b-m	39.33 C-F
B18	11.89c-m	7.85 w-J	3.57 f-m	2.53 r-D	4.75 c-g	2.75 l-n	3.74 f-p	2.21 C-I	63.5 f-w	51.92 q-D
B19	10.76 e-u	6.98 A-J	3.48 f-n	1.94 A-D	4.25 d-h	2.75 l-n	3.53 h-t	2.29 A-I	60.92 i-w	38.42 C-F
B20	10.69 f-v	6.65 C-J	3.91 d-k	2.04 z-D	4.75 c-g	2.75 l-n	3.8 e-p	2.1 D-I	69.75 d-o	39.42 C-F
B24	11.02 e-r	5.68 I-J	3.54 f-n	1.92 A-D	3.92 f-j	2.58 l-n	3.69 g-q	2.23 B-I	65.83 e-s	37.33 C-F
B25	11.44 d-o	7.27 y-J	3.35 g-s	2.36 v-D	3.83 g-k	2.5 m-n	3.81 e-p	1.99 E-I	60.17 j-w	47.92 u-F
B26	12.68 b-i	6.47 C-J	4 b-j	1.94 A-D	4.17 e-i	2.33m-n	3.9 e-o	2.06 D-I	68.58 e-q	35.42 D-F
B27	11.28 d-q	6.4 C-J	3.97 c-j	1.78 B-D	4.17 e-i	2.75 l-n	3.74 f-p	2.32 A-I	67.67 e-r	38.08 C-F
B28	11.82 c-n	6.73 B-J	3.88 d-k	2.08 z-D	4.25 d-h	2.25 n	3.99 e-m	2.2 C-I	74.33 b-l	42 y-F
B29	10.42g-w	5.86 H-J	3.42 f-p	1.89 A-D	4.25 d-h	2.67 l-n	3.75 f-p	1.95 F-I	63.67 f-v	40.17 C-F
S-1	11.92 c-l	7.97 v-I	3.79 e-l	2.45 u-D	4.5 c-g	2.33m-n	4.04d-m	2.15 D-I	74.25 b-l	48.75 t-F
S-3	12.31 b-k	7.43 y-J	4.26 a-f	2.42 u-D	4.83 c-f	2.83 l-n	4.2 d-i	2.22 C-I	78.33 a-g	50.08 s-F
S-4	10.89 e-s	8.23 s-I	3.41 f-q	2.43 u-D	4.58 c-g	2.92 k-n	3.74 f-p	2.45 y-H	62.83 g-w	53.83 n-C
S-6	12.21 b-k	8.58 q-H	4.14 a-h	2.7 n-A	4.67 c-g	3.17 j-n	3.73 g-p	3.1 n-A	70.92 d-m	58.67 l-y
S-7	11.82 c-n	7.59 x-J	4.04 a-j	2.34 v-D	4.33 c-h	2.83 l-n	3.93 e-o	2.59 u-H	75.67 a-k	50.58 s-E

S-12	13 a-h	7.41 y-J	4 b-j	2.08 z-D	5.17 b-d	3.25 i-m	4.06d-m	2.1 D-I	76.75 a-j	41.92 z-F
S-14	9.61 k-A	8.08 t-I	3.32 h-t	2.36 v-D	4.83 c-f	2.83 l-n	3.76 e-p	2.46 x-H	64.33 f-u	51.75 r-D
D1	12.49 b-j	9.05 n-D	3.79 e-l	2.55 q-C	4.25 d-h	3.08 j-n	3.94 e-o	2.5 v-H	74.17 b-l	53.42 o-C
D4	11.27 d-q	8.38 r-I	3.82 e-l	2.61 o-B	5.25 b-c	3.5 h-l	3.89 e-p	3.32 k-x	70.82 d-m	52.33 q-C
D5	14.98 ab	7.64 x-J	4.78 a-c	2.29 w-D	6.33 a	2.75 l-n	5.33 ab	2.17 C-I	87.83 a-c	46.92w-F
E3	12.81 b-h	8.06 u-I	4.86 ab	2.4 v-D	6.33 a	2.83 l-n	5.39 a	2.13 D-I	88.08 ab	48.17 u-F
E4	12.96 a-h	8.43 r-I	3.84 d-l	2.49 t-D	5.25 b-c	2.83 l-n	3.83 e-p	2.31 A-I	73.25 b-m	52.75 p-C
E6	12.29 b-k	8.37 r-I	3.36 g-r	2.49 s-D	4.42 c-h	2.92 k-n	3.9 e-p	2.68 t-G	61.25 h-w	52.58 p-C
F1	10.46g-w	7.49 x-J	3.42 f-p	2.08 z-D	4.83 c-f	2.42m-n	4.05d-m	2.34 A-I	62.42 g-w	41.9 z-F
F2	9.77 j-z	8.69 o-F	3.19 j-v	2.59 o-B	4.92 b-e	2.5 m-n	4.2 d-j	2.32 A-I	60.92 i-w	47.25 v-F
P12	12.05 c-l	8.28 r-I	3.8 e-l	2.1 z-D	5.17 b-d	2.33m-n	4.38 c-h	2.4 z-I	72.33 b-m	39.75 C-F
Inh1	13.94 a-d	7.79 w-J	4.21 a-g	2.12 y-D	4.67 c-g	2.83 l-n	4.61 a-f	2.85 q-E	73.33 b-m	39.75 C-F
Inh2	13.99 a-d	9.14 m-C	3.88 d-k	2.48 t-D	5.83 ab	2.67 l-n	4.51 b-g	2.8 r-F	69.25 e-p	49.75 s-F
Inh3	12.11 c-l	8.03 u-I	3.98 c-j	2.15 x-D	5.25 b-c	2.67 l-n	4.15 d-l	2.86 q-D	72.67 b-m	43.08 x-F
Inh5	14.58 a-c	10.24 h-x	4.5 a-e	2.49 t-D	5 b-e	2.75 l-n	4.19 d-k	2.8 r-F	81.25 a-e	51.92 q-D
Inh6-1	9.79 j-z	9.67 k-A	3.11 k-w	2.3 w-D	4.92 b-e	2.58 l-n	3.55 h-s	2.58 u-H	59.58 k-x	47 v-F
Inh8	13.53 a-e	8 u-I	4.07 a-i	2.03 z-D	5.17 b-d	2.5 m-n	4.86 a-d	2.69 s-F	77.75 a-h	42.92 x-F
AB3	12.28 b-k	7.62 x-J	3.54 f-n	2.02 z-D	5.01 b-e	2.75 l-n	4.22 d-i	2.7 r-F	62.98 g-w	40.67 B-F
AB4	13.26 a-f	7.9 w-I	3.71 e-m	1.98 A-D	4.92 b-e	2.33m-n	4.05d-m	2.71 r-F	70.75 d-m	39.33 C-F
AB5	15.73 a	8.88 o-E	3.84 d-l	2.28 w-D	4.92 b-e	2.33m-n	3.89 e-p	2.53 v-H	71.17 c-m	49.83 s-F
Ph4	11.69 d-n	7.8 w-J	3.27 i-u	2.11 z-D	4.83 c-f	2.38m-n	4.19 d-j	2.85 q-E	57.15 m-B	41.27 A-F
**Mean**	**11.78A**	**7.47B**	**3.79A**	**2.21B**	**4.78A**	**2.77B**	**3.95A**	**2.39B**	**69.54A**	**45.52B**

Values are the means of three replicates. Means sharing at least one letter are not significantly different according to Tukey's HSD test at P ≤ 0.05. Hyphenated letter ranges represent all consecutive Tukey grouping letters within the range (e.g., C–H = C, D, E, F, G, H).

Table S4. Mean comparison of grain yield (GY), biological yield (BY), harvest index (HI), and irrigation water use efficiency (IWUE) of wheat inoculated with different *Bacillus st*rains (BS) under full irrigation (FI) and deficit irrigation (DI).

BS	GY (kg m^{-2})		BY (kg m^{-2})		HI (%)		IWUE (kg m^{-3})	
	FI	DI	FI	DI	FI	DI	FI	DI
Control	462.72 f-v	350.8 u-E	1426.33 f-n	904.87 y-G	34.79 b-l	38.87 a-l	1.29 s-C	1.88 c-l
D2	640.63 a-c	438.1 h-z	1898.73 a-c	1207 n-x	33.57 c-l	36.65 a-l	1.68 e-w	2.35 ab
Inh10	502.05 e-n	376.55 q-E	1522.62 f-m	954.77 x-G	33.15 d-l	40.61 a-h	1.31 s-C	2.02 b-g
M1-1	524.77 c-k	357.33 u-E	1605 d-j	996 v-E	32.89 e-l	36.71 a-l	1.37 o-C	1.92 b-k
A2	674.63 a	507.53 e-m	2134.77 a	1614.98 c-j	31.68 h-l	31.22 j-l	1.77 d-r	2.72 a
SS3	591.17 a-e	417.03 i-B	1863.12 a-d	1042.18 t-C	31.61 i-l	40.37 a-i	1.55 h-A	2.24 b-c
B2	402.53 l-C	312.3 A-F	1284.95 l-v	766.08 C-G	31.13 k-l	40.66 a-g	1.05 C	1.68 e-w
B3	481.17 e-t	275.02 D-F	1303.13 l-t	704.02 F-G	36.98 a-l	39.02 a-l	1.26 v-C	1.48 k-C
B4	442.9 h-y	299.92 B-F	1269.78m-w	750 D-G	35 a-l	39.79 a-k	1.16 z-C	1.61 f-y
B6	531.58 c-k	259.38 E-F	1543.5 f-m	688.08 G	34.86 a-l	37.58 a-l	1.39 n-C	1.39 n-C
B7	439.85 h-z	294.1 C-F	1291.8 l-u	773.83 C-G	33.88 b-l	37.88 a-l	1.15 z-C	1.58 g-z
B8	464.72 f-v	321.97 z-F	1305.68 k-t	822.17 y-G	35.67 a-l	39.61 a-l	1.21 y-C	1.73 d-t
B9	484.68 e-s	315.5 A-F	1347.1 j-r	848.45 y-G	36.78 a-l	36.85 a-l	1.26 v-C	1.69 e-v
B10	533.57 c-j	310.57 A-F	1620.7 c-j	841.78 y-G	32.82 e-l	36.56 a-l	1.4 n-C	1.67 e-w
B11	425.1 i-A	319.2 z-F	1297.58 l-t	815.63 y-G	33.41 c-l	39.54 a-l	1.11 A-C	1.71 e-u
B14	470.38 f-u	216.12 F	1375.92 h-o	707.73 E-G	34.86 a-l	32.99 e-l	1.22 x-C	1.16 z-C
B15	501.65 e-o	300.1 B-F	1478.25 f-n	843.87 y-G	34.12 b-l	35.18 a-l	1.31 s-C	1.61 f-y
B18	492.67 e-q	374.45 q-E	1391.02 g-n	943.22 x-G	35.13 a-l	40.11 a-j	1.28 t-C	2.01 b-g

B19	490.83 e-q	310.72 A-F	1309.72 k-t	823 y-G	37.27 a-l	38.43 a-l	1.28 u-C	1.67 e-w
B20	535.38 c-i	315.25 A-F	1560.88 e-l	798.58 z-G	34.01 b-l	41.18 a-e	1.4 n-C	1.69 e-v
B24	481.57 e-t	308.67 A-F	1360.33 i-q	750.67 D-G	34.82 b-l	42.24 a-c	1.26 v-C	1.66 e-x
B25	446.18 h-y	356.52 u-E	1360.48 i-q	982.83 w-F	33.16 d-l	36.21 a-l	1.17 y-C	1.91 b-l
B26	508.17 e-m	274.1 D-F	1524.9 f-m	758.67 C-G	33 e-l	36.85 a-l	1.33 r-C	1.47 l-C
B27	519.98 c-l	280.27 D-F	1546.22 f-m	767.67 C-G	33.6 c-l	36.55 a-l	1.36 p-C	1.5 j-B
B28	528.67 c-k	337.27 x-E	1520.92 f-m	782.97 B-G	34.16 b-l	42.78 ab	1.38 o-C	1.81 c-o
B29	488.45 e-r	321.92 z-F	1389.02 g-n	833.1 y-G	35.24 a-l	38.67 a-l	1.27 u-C	1.73 d-t
S-1	507.97 e-m	354.4 u-E	1571.85 e-l	934.73 x-G	32.19 f-l	37.73 a-l	1.33 r-C	1.9 c-l
S-3	576.52 a-g	364.62 s-E	1677.32 c-g	946.07 x-G	34.33 b-l	38.44 a-l	1.51 j-B	1.96 b-i
S-4	457.3 g-x	379.97 p-E	1342.92 j-r	1045.58s-C	34.35 b-l	36.28 a-l	1.2 y-C	2.04 b-f
S-6	514.82 e-l	412.85 k-C	1555.57e-m	1084.1 p-z	32.96 e-l	38.03 a-l	1.35 r-C	2.22 b-c
S-7	519.35 d-l	386.63 n-D	1645 c-i	995 w-E	31.26 j-l	39.13 a-l	1.36 q-C	2.08 b-e
S-12	524.67 c-k	347.78 v-E	1694.83 b-f	901.98 y-G	31.52 i-l	37.77 a-l	1.37 o-C	1.87 c-m
S-14	481.12 e-t	380.98 p-D	1369.75 i-p	986.92 w-F	34.76 b-l	38.38 a-l	1.23 w-C	2.04 b-f
D1	500.42 e-p	386.23 n-D	1594.38 d-k	1064.33 r-B	31.61 i-l	36.54 a-l	1.31 s-C	2.07 b-e
D4	525.58 c-k	402.43 l-C	1614.28 c-j	1045.67s-C	32.75 e-l	38.51 a-l	1.38 o-C	2.16 b-d
D5	638.3 a-d	375.23 q-E	1983.78 ab	1072.83q-A	32.08 f-l	35.32 a-l	1.67 e-w	2.01 b-g
E3	662.52 a	357.43 u-E	1876.58 a-d	936.67 x-G	35.13 a-l	38.76 a-l	1.73 d-s	1.92 b-k
E4	528.1 c-k	389.42m-D	1660.7 c-h	1025.57 t-D	31.46 i-l	38.83 a-l	1.38 o-C	2.09 b-e
E6	466.12 f-v	381.28 o-D	1332.17 j-s	975.25 x-G	34.44 b-l	39.76 a-k	1.22 x-C	2.05 b-f
F1	458.07 g-w	342.03 w-E	1299.63 l-t	883.68 y-G	35.27 a-l	39.53 a-l	1.2 y-C	1.84 c-n
F2	459.48 g-w	373.15 q-E	1342 j-r	1001.83v-D	34.76 b-l	37.38 a-l	1.2 y-C	2 b-g
P12	522.68 c-l	329.12 y-F	1603.1 d-j	781.05 B-G	32.45 e-l	42.04 a-d	1.37 o-C	1.77 d-r
Inh1	533.05 c-k	334.62 y-F	1535.85 f-m	767.7 C-G	35.03 a-l	43.81 a	1.39 n-C	1.8 c-q
Inh2	498.62 e-p	369.1 r-E	1647.65 c-i	1002.73u-D	30.71 l-l	37.6 a-l	1.3 s-C	1.98 b-h
Inh3	509.97 e-m	336.35 y-F	1547.38 f-m	855.8 y-G	33.27 c-l	39.27 a-l	1.33 r-C	1.81 c-p
Inh5	580.95 a-f	376.22 q-E	1842.32 b-e	1094.5 o-y	31.64 h-l	35 a-l	1.52 i-B	2.02 b-g
Inh6-1	413.43 j-C	352.92 u-E	1300.33 l-t	950.55 x-G	31.83 g-l	37.21 a-l	1.08 B-C	1.89 c-l
Inh8	547.98b-h	348.85v-E	1648.52 c-i	850.63 y-G	33.65 c-l	40.84 a-f	1.43 m-C	1.87 c-m
AB3	481.45 e-t	314.32 A-F	1313.62 k-t	785.78 A-G	37.39 a-l	40.15 a-j	1.26 v-C	1.69 e-v
AB4	530.67c-k	322.2 z-F	1458 f-n	801.98 z-G	36.74 a-l	39.95 a-k	1.39 o-C	1.73 d-t
AB5	529.58c-k	361.33 t-E	1481.02 f-n	997 v-E	35.89 a-l	37.75 a-l	1.39 o-C	1.94 b-j
Ph4	458.47g-w	335.23 y-F	1371.27 i-p	892.6 y-G	33.85 b-l	37.61 a-l	1.2 y-C	1.8 c-q
**Mean**	**509.48A**	**345.49B**	**1516.20A**	**911.59B**	**33.83B**	**38.36A**	**1.33B**	**1.85A**

Values are the means of three replicates. Means sharing at least one letter are not significantly different according to Tukey's HSD test at P ≤ 0.05. Hyphenated letter ranges represent all consecutive Tukey grouping letters within the range (e.g., C–H = C, D, E, F, G, H).

Table S5. Eigenvalue, cumulative variability, and factor loadings of the first two principal components (PCs) for different growth- and yield-related traits and plant growth–promoting traits under full irrigation (FI) and deficit irrigation (DI).

**Traits**	**FI**		**traits**	**DI**	
	**PC1**	**PC2**		**PC1**	**PC2**
PH	0.076	**0.360**	PH	0.042	**0.399**
TN	-0.005	0.044	TN	-0.088	-0.075
LN	0.023	**0.360**	LN	-0.003	**0.252**
TLA	**0.242**	-0.154	TLA	**0.229**	0.122
TFW	**0.187**	0.085	TFW	**0.199**	**0.291**
TDW	**0.236**	-0.193	TDW	**0.239**	0.120
SpN	**0.203**	-0.001	SpN	**0.116**	**-0.141**
SpW	**0.207**	0.016	SpW	0.153	**0.262**
SN	**0.239**	-0.173	SN	**0.243**	0.061
GY	**0.241**	-0.180	GY	**0.245**	0.105
BY	**0.259**	-0.156	BY	**0.250**	0.048
HI	**-0.134**	0.015	HI	-0.091	0.096
IWUE	**0.241**	-0.180	IWUE	**0.245**	0.105
IAA	**0.202**	-0.070	K	0.009	-0.107
NH3	0.098	-0.242	Sid	-0.080	-0.091
Eigenvalue	3.445	2.023			
Variability (%)	0.396	0.136			
Cumulative	0.396	0.532			

PH, plant height (cm); TN, tiller number per plant; LN, leaf number per plant; TLA, total leaf area (cm^2 $plant^{-1}$); TFW, total fresh weight (g $plant^{-1}$); TDW, total dry weight (g $plant^{-1}$); SpN, spike number per plant; SpW, spike weight (g $plant^{-1}$); SN, seed number per plant; GY, grain yield (kg m^{-2}); BY, biological yield (kg m^{-2}); HI, harvest index (%); IWUE, irrigation water-use efficiency (kg m^{-3}); IAA, indole-3-acetic acid; NH_3, ammonia; Sid, siderophore production; and K, potassium-solubilizing activity.
